# Halogenases: a palette of emerging opportunities for synthetic biology–synthetic chemistry and C–H functionalisation

**DOI:** 10.1039/d0cs01551b

**Published:** 2021-08-09

**Authors:** Charlotte Crowe, Samuel Molyneux, Sunil V. Sharma, Ying Zhang, Danai S. Gkotsi, Helen Connaris, Rebecca J. M. Goss

**Affiliations:** School of Chemistry, and BSRC, University of St Andrews, North Haugh St Andrews KY16 9ST UK rjmg@st-andrews.ac.uk

## Abstract

The enzymatic generation of carbon–halogen bonds is a powerful strategy used by both nature and synthetic chemists to tune the bioactivity, bioavailability and reactivity of compounds, opening up the opportunity for selective C–H functionalisation. Genes encoding halogenase enzymes have recently been shown to transcend all kingdoms of life. These enzymes install halogen atoms into aromatic and less activated aliphatic substrates, achieving selectivities that are often challenging to accomplish using synthetic methodologies. Significant advances in both halogenase discovery and engineering have provided a toolbox of enzymes, enabling the ready use of these catalysts in biotransformations, synthetic biology, and in combination with chemical catalysis to enable late stage C–H functionalisation. With a focus on substrate scope, this review outlines the mechanisms employed by the major classes of halogenases, while in parallel, it highlights key advances in the utilisation of the combination of enzymatic halogenation and chemical catalysis for C–H activation and diversification.

## Abstract 2

An overview of enzymatic tools for generation of carbon–halogen bonds and their use in enabling selective C–H functionalisation through combination of enzyme and chemo catalysis.

## Graphical abstract

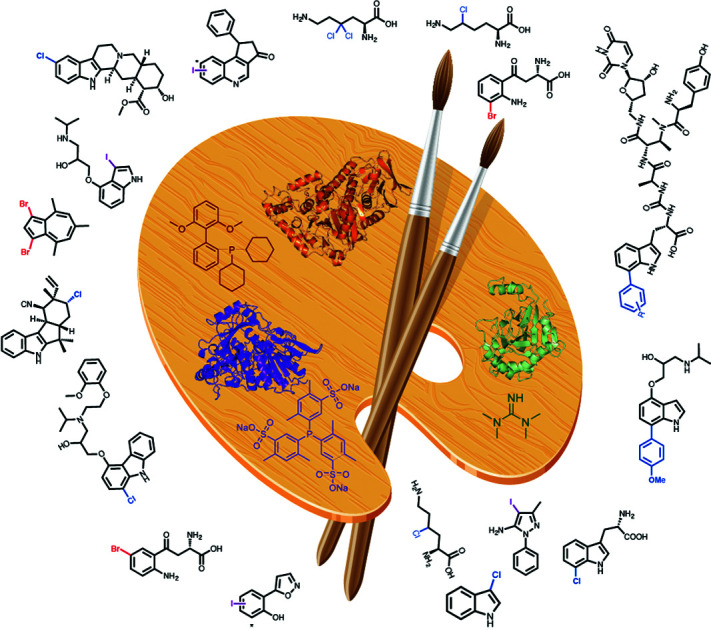

## Introduction

**Fig. 1:**
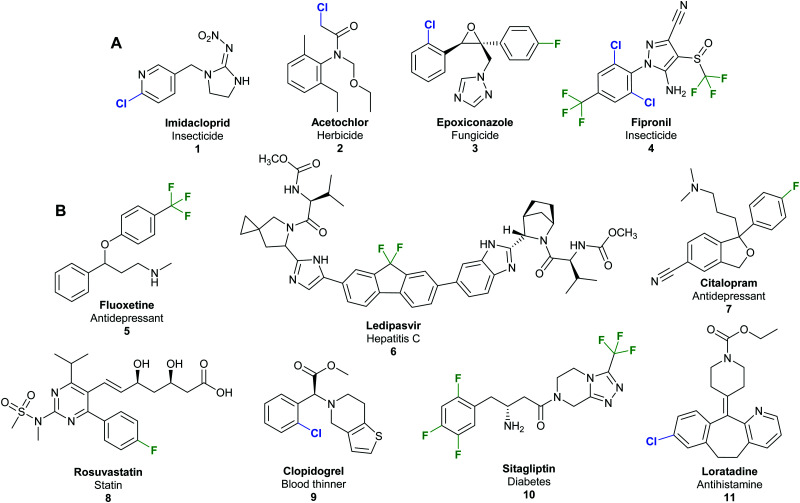
Examples of commercially important and structurally diverse organohalides. (A) Examples of top-selling halogenated agrochemicals include imidacloprid **1**, biggest selling insecticide 2011 sales of 1.09 billion USD per annum;^2^ acetochlor **2**, herbicide in the top 10 best-selling agrochemicals in 2003;^16^ epoxiconazole **3**, a triazole based fungicide with sales of 215 million USD in 2001;^17^ and fipronil **4**, a pyrazole based insecticide with sales of 270 million USD in 2001.^17^ (B) Examples of top-selling halogenated pharmaceuticals include antidepressants fluoxetine **5** (Prozac®)^17^ and citalopram **7** (Celexa®);^18^ anti-hepatitis C agent ledipasvir **6**;^5^ statin rosuvastatin **8**;^19^ the blood thinner clopidogrel **9**;^16^ the type 2 diabetes treatment sitagliptin **10**;^20^ and antihistamine loratadine **11**.^6^

Halogenated organic compounds play an important role in society. In the agrochemical sector, a staggering 96% of herbicides, fungicides, insecticides, acaricides and nematicides produced since 2010 contain halogen atoms.^1^ Such compounds are valuable commodities; for example, the top-selling insecticide imidacloprid (Fig. 1A) **1** had sales of 1.09 billion USD in 2011 alone,^2^ and in 2013, 50% of marketed top drugs contain halogen atoms.^3,4^ The fluorinated hepatitis C ledipasvir/sofosbuvir combination (Harvoni®) **6** was the second best-selling blockbuster pharmaceutical in 2016 with USA sales revenues of 4.9 billion USD (Fig. 1B),^5^ whilst, in the same year, US sales of the chlorinated antihistamine loratadine (Claritin®) **11** exceeded 2 billion USD.^6^ An economic report analysing the annual sales of the 100 top selling pharmaceuticals reveals that 12.9% of the final active pharmaceutical ingredient (API) is chlorinated or brominated, whilst a further 62.7% require chlorination at some stage in their manufacture.^7^

The incorporation of a carbon–halogen bond is a regularly employed strategy in medicinal and agro-chemistry. Due to bond polarisation and the ability to form highly directional halogen bonds to electron-rich oxygen, nitrogen, sulfur and aromatic rings, halogen incorporation may be used to enhance stability, and increase lipophilicity which in turn can influence bioavailability and modulate compound bioactivity.^8^ Beyond these applications, series of fluorinated and iodinated compounds are used in radiotherapy and biomedical imaging.^9,10^ Halogenation is also central to molecule construction and manufacture. The reactivity and chemical orthogonality of organohalides enables many selective transformations including, most notably, cross-coupling and substitution chemistries. Synthetically, the generation of organohalides is currently achieved through the addition of molecular halogens, hydrohalogenation, or the use of reagents enabling nucleophilic substitution and electrophilic aromatic substitution [EAS]. These reactions are generally accompanied by environmental and selectivity challenges due to the toxic, corrosive and reactive nature of the reagents used, and the propensity to generate complex product mixtures.^11^ Whilst recent advances in homogenous catalyst design has meant more selective catalytic approaches towards halogenation are known, such catalysts can be complex, costly and time consuming to synthesise.^12^

Though naturally occurring organohalides were initially speculated to be nothing more than a biological curiosity, artefact, or to be anthropogenic in origin, today, over 5000 halogenated natural products are known, with genes encoding halogenases identified across all kingdoms of life.^13^ These represent a resource for greener and often highly regioselective halogenation. An increased appreciation of mechanisms underpinning enzymatic halogenation has evolved over the past 25 years, and may broadly be classified as electrophilic, nucleophilic or radical halogenation. Whilst a good level of understanding of the enzymatic active sites and structural requirements of these enzymes exists, a number of fundamental details remain to be elucidated.

This review overviews the mechanisms of the different classes of halogenases, the natural context of the halogenases, their biocatalytic applications, and explores their powerful potential for use in the hyphenation of Synthetic Biology and Synthetic Chemistry (SynBio–SynChem) enabling C–H activation and diversification.

### 1. Mechanisms for biocatalytic halogenation

Enzymatic halogenation is classified mechanistically in accordance with whether the halogen is introduced by an electrophilic, nucleophilic, or radical halide species, with the vast majority of the enzymes that have been discovered so far, being electrophilic halogenases, mediating oxidation of the halogen to generate a reactive electrophilic species. There is further subdivision within the broader halogenase categories, based on the exact catalytic species and cofactor which enables halogen oxidation.

#### 1.1 Electrophilic (X^+^) halogenation

##### 1.1.1 Haloperoxidases

**Scheme 1:**
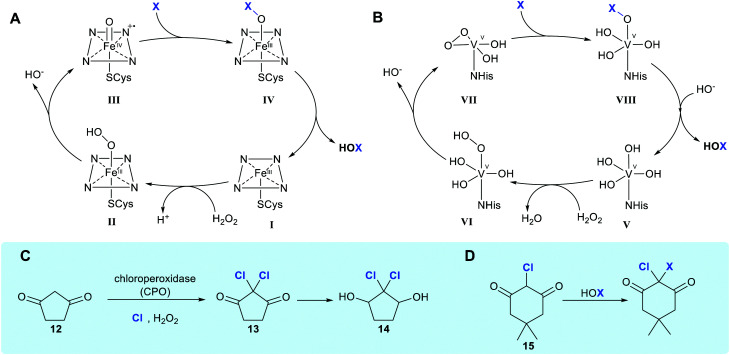
Haloperoxidase HOX generation and utilisation. (A and B) Biocatalytic cycle of heme iron-dependent and vanadium-dependent haloperoxidases, respectively, showing the release of freely diffusible hypohalous acid (NB the order of events in B remains unclear, with the protonation state of VII being ambiguous).^21^ (C) Chlorination of 1,3-cyclopentanedione **12** by HOCl in the presence of a heme iron-dependent chloroperoxidase (CPO) and hydrogen peroxide suggested to occur in caldariomycin **14** biosynthesis.^15^ (D) The reaction of monochlorodimedone (MCD) **15** with HOX, the benchmark assay for haloperoxidase activity.^22^

Haloperoxidases are defined as enzymes that utilise hydrogen peroxide and chloride, bromide or iodide to generate HOX. These enzymes are named in accordance with the most electronegative halogen for which they can affect oxidation. Haloperoxidases are generally the least regioselective of the enzymes enabling halogenation; catalysing the generation of free hypohalous acid, which in all but a few cases, is released by the enzyme. The earliest reported studies of halogenation enabled by an enzyme date back to 1959, when Lowell Hager discovered a haloperoxidase (HPO) from the fungus *Calariomyces fumago*.^14^ The enzyme that Hager had utilised as part of a lysate was named chloroperoxidase or (CPO) and was shown to be capable of affecting dichlorination of 1,3-cyclopentanedione **12**. It has thus been postulated to be involved in the biosynthesis of the antibiotic caldariomycin **14** (Scheme 1C).^15^ CPO may be described more specifically as a heme iron-dependent haloperoxidase, a subset of haloperoxidases that contain a heme-iron cofactor coordinated to an axial cysteine ligand within the enzyme. Hydrogen peroxide binds axially to heme-Fe(iii) in its resting state to form a ˙^+^heme-Fe(iv)=O intermediate, denoted compound I.^23^ Addition of halide to the ferryl oxygen produces a heme-Fe(iii)–OX species (Scheme 1A, IV). Free hypohalous acid, HOX, is generated which reacts with an electron-rich substrate (Scheme 1A).^24^ Generation of electrophilic X^+^ allows halogenation of electron-rich compounds by electrophilic aromatic substitution, generally at the most reactive site(s) within the substrate. CPO has been used biocatalytically to mediate the chlorination, bromination or iodination of a variety of electron-rich substrates (see Section 2), however, as it lacks a site to position the substrate, no regiocontrol beyond that conferred by the electronics of the substrate is observed.

The vanadium-dependent haloperoxidases (V-HPOs) operate by an analogous mechanism, again generating and, mostly releasing, free hypohalous acid. This subcategory of haloperoxidase is widely found in algae, fungi and bacteria, and utilises a vanadium cofactor bound by an axial histidine ligand.^25^ In the resting state, the cofactor is in a trigonal bipyramidal configuration (Scheme 1B, V), a distal H_2_O_2_ displaces two hydroxyl ligands giving rise to formation of the known η^2^-peroxido complex (Scheme 1B, VII), with the distorted square pyramidal geometry. Though the exact order of events and level of protonation here is not known, a postulated catalytic cycle is shown. A lysine residue hydrogen bonded to an equatorial V–O oxygen activates the peroxide complex for X^−^ attack and a return to the trigonal bipyramidal geometry (Scheme 1B, VIII) is observed. The resulting V–OX species undergoes hydrolysis to generate electrophilic X^+^ in the form of free HOX.^26^ Electrophilic aromatic substitution occurs, as in the case with heme-iron haloperoxidases.^27^ A notable difference with the heme iron-dependent biocatalytic cycle is that the vanadium centre maintains its oxidation state throughout the process.^28^

Haloperoxidases had initially been considered to lack significant enzyme-mediated selectivity due to the release of freely diffusible hypohalous acid. The free electrophilic species allows halogenation at the most electron-rich carbon, and a mixture of mono-, di- and tri-substituted products can often be observed. Detection of the free HOX through reaction with nucleophilic monochlorodimedone (MCD) **15** has previously been used to assay for haloperoxidase activity (Scheme 1D).^29^ However, for a number of vanadium-dependent haloperoxidases, substrate, regio- and even stereo-specific halogenation is evident, as noted in the biosynthesis of napyradiomycins **76**,^30^ and merochlorins **77**, **78**.^31^ (Scheme 5). Currently, the exact mechanistic rationale for this specificity is not known. Here, free HOCl is notably not detected in assay with MCD.^31^ It is thus postulated that residues within the enzyme might transiently support the electrophilic HOCl, whilst the electron-rich substrate is appropriately and proximally positioned. Moore invokes the involvement of an active site lysine forming a haloamine, in a manner analogous to the FDHs.^31,32^ Though NapH1 from napyradiomycin biosynthesis is associated with the first chlorination event, this enzyme is also capable of mediating nonspecific bromination through generation and release of HOBr. Greater understanding of the mechanism enabling selective halogenation by these series of enzymes would be valuable, potentially enabling informed discovery of further enzymes with biotechnological potential.

##### 1.1.2 Flavin-dependent halogenases

The haloperoxidases remained the only known halogenases for several decades until in 1995, the first of a second series of halogenases, the flavin-dependent halogenases (FDHs), Chl from 7-chlortetracycline **73** biosynthesis in *Streptomyces aureofaciens* was revealed in a study by Dairi.^33^ The enzyme was implicated as being involved in chlorination with knockouts of the gene encoding Chl lacking halogenated product. However, from these initial studies, it was not fully apparent that this new series of enzymes were flavin-dependent, only a partial protein sequence lacking 100 amino acids at the N-terminal end including the flavin binding site. *In vitro* analysis of the tryptophan 7-halogenase PrnA, by van Pée and co-workers, demonstrated the powerful regioselectivity of this new class of halogenase and the requirement for both flavin and molecular di-oxygen.^34,35^

**Scheme 2:**
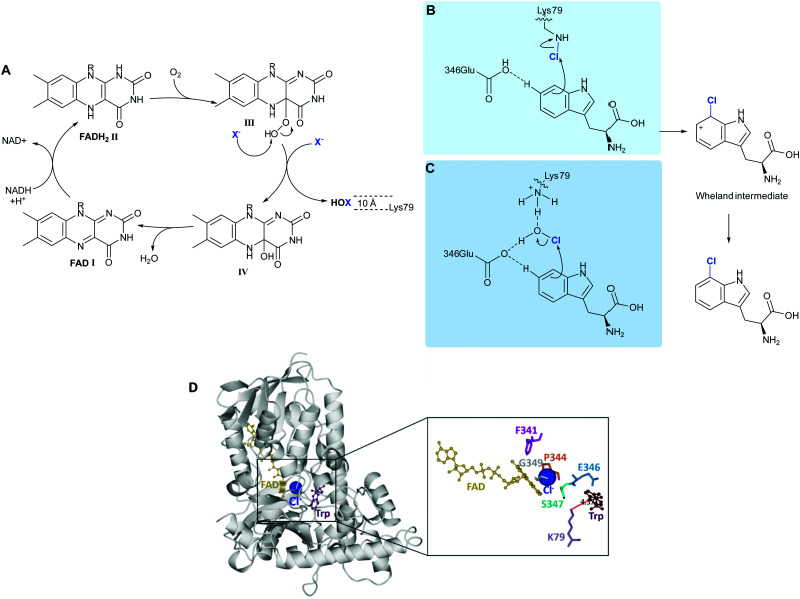
FDH-mediated electrophilic halogenation. (A) Biocatalytic cycle of a flavin-dependent halogenase, showing the release of hypohalous acid. HOX is channelled through a ∼10 Å tunnel. The regioselectivity of the electrophilic aromatic substitution is postulated to be achieved by: (B) a lysine residue, at the end of the tunnel that reacts with HOX to form a haloamine intermediate,^43^ and (C) lysine and glutamine, acting in concert to position HOX and further electronically activate the tryptophan.^39^ (D) Extrapolation of the crystal structure of PrnA showing how Lys79 is positioned over C7 of the indole ring mediating regioselective halogenation.

The FDHs can be separated into those acting on free substrates (variant A), and those requiring their substrate to be covalently tethered to a carrier protein *via* a phosphopantatheine linker (variant B).^36^ The most extensively investigated and engineered FDHs are the tryptophan halogenases, variant A FDHs enabling regioselective halogenation of tryptophan at the 5, 6 or 7 positions. The first structural insights as to how FDHs mediate their exquisite regioselectivity were revealed by van Pée and Naismith.^37^ However, 15 years on and debate still remains as to the exact way in which these enzymes function. Details as to which residues within the active site participate may vary from one enzyme to another. The mechanisms proposed by Walsh^38^ and van Pée^39^ for the flavin dependent tryptophan halogenase are presented in Scheme 2B and C.

Reduced flavin adenine dinucleotide (FADH_2_), (Scheme 2A, I) and dioxygen in the presence of chloride, bromide or iodide are utilised by these enzymes to catalyse C–H to C–X. Freely diffusible FAD binds to a conserved GxGxxG motif. The reduced cofactor, FADH_2_, (Scheme 2A, II) reacts with molecular oxygen to form FAD(C4α)–OOH (iii), which subsequently reacts with a nucleophilic halide releasing HOX and FAD(C4α)–OH (Scheme 2A, IV).^40^ Unlike haloperoxidases, the HOX generation and electrophilic aromatic substitution (EAS) events are spatially separated by a ∼10 Å tunnel. At the end of this tunnel is a conserved lysine, proposed by Walsh to react with the HOX thereby forming a haloamine intermediate, guiding the regiochemistry of halogenation. (Scheme 2B).^38^ Mutation of this lysine residue to alanine, across a series of halogenases including RebH, PrnA, and Rdc2, results in loss of activity.^41^ In a subtly different mechanism, van Pée invokes a proximal glutamate residue, as well as the lysine residue binding to HOX, thereby activating it and positioning it (Scheme 2C).^39^ In both models, the electrophilic halogenating species is directed towards the substrate, promoting a regioselective EAS reaction. The exact mechanism and halogenating species remain elusive. Mutation of this active site glutamate to glutamine in PrnA results in a drop in rate by two orders of magnitude.^37^ This glutamate is not present in the more electron-rich phenol halogenases. A different proposed role for this active site glutamate, invoked in deprotonation of the Wheland intermediate indole benzo ring in the tryptophan halogenases, is that it perhaps plays a role in reducing the activation energy of halogenation of these less electron-rich species.^41^ Notably no kinetic isotope effect is observed for halogenation of tryptophan by RebH.^42^

**Fig. 2:**
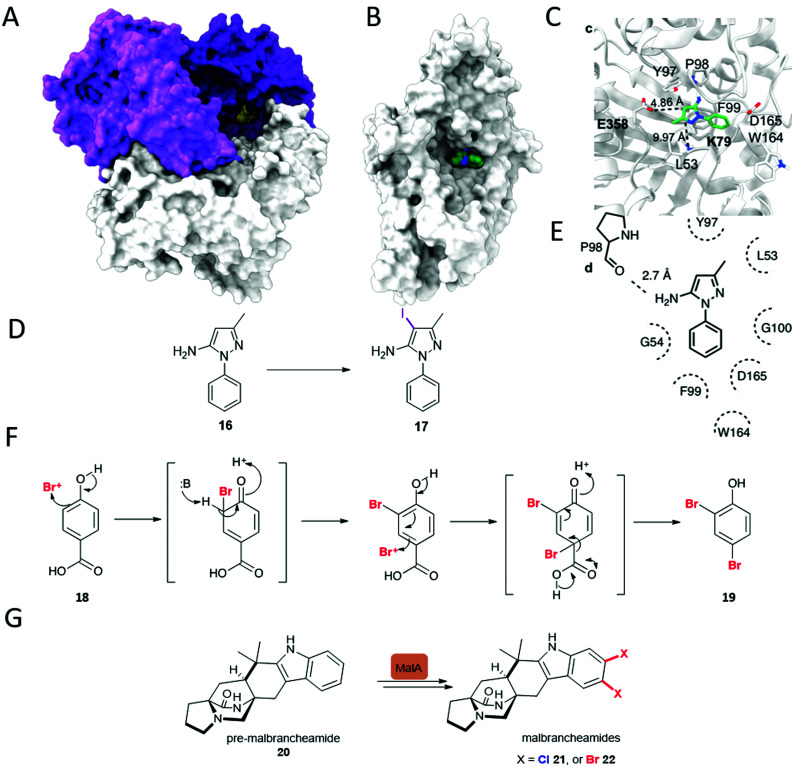
Unusual FDHs. (A) Trimer of VirX1, the first FDH with a preference for iodination, and the first halogenase from a virus showing the large and accessible active site, with a bulky substrate **16** modelled in the active site. (B) View of a subunit of VirX1, again with substrate **16** modelled into the active site, and embedded in a hydrophobic cleft. (C) Key hydrogen bonds between VirX1 and **16** as predicted by structural modelling. (D) Regiospecific iodination of **16** by VirX1.^47^ (E) Model of compound **16** in the active site of VirX1. (F) Decarboxylative bromination of **18** observed for Bmp5.^48^ (G) Iterative dihalogenation in malbrancheamides **21** and **22** biosynthesis, by an unusual zinc-containing FDH.^46^

These FDHs share a similar flavin binding domain to the flavin-dependent monoxygenases, and though most utilise free and diffusible FAD, which is converted to FADH_2_, variants exist where the flavin is covalently bound to the enzyme, such as in the case of CmlS involved in chloramphenicol biosynthesis.^44^ Naturally, most FDHs are usually provided the reduced flavin by a partner flavin reductase that utilises NADH (nicotinamide adenine dinucleotide) and FAD to generate reduced FADH_2_.^35^ The partner flavin reductase can be readily substituted for any other capable of producing FADH_2_. Single component FDHs, that have this reductase function present within the one protein, also exist; the first discovered example of such an enzyme was Bmp5.^45^ This enzyme shows other notable differences to all previously studied FDHs; in analogy to the decarboxylative hydroxylation observed for a single component monooxygenase, decarboxylative dibromination of **18** occurs (Fig. 2F).^45^ Other intriguing variations of the FDHs also exist, recently, the first of a new class of zinc-binding FDHs, MalA from the biosynthesis of the malbrancheamides **21** and **22** (Fig. 2G) was reported. This remarkable enzyme is capable of mediating the late stage dichlorination of 5, and 6 positions of the indole benzo ring of pre-malbrancheamide **20**, a complex prenylated indole alkaloid. In the first step, positions 5 and 6 appear to be halogenated with comparable rates, perhaps due to increased electron density, the second halogenation even occurs far more quickly. Within the enzyme, the active site glutamate is seen proximal to the NH of the indole. It is clearly implicated in deprotonating this moiety, rendering the substrate more nucleophilic, and whilst mutation of this glutamate to aspartate results in low but observable activity, mutation to glutamine renders the enzyme inactive.^46^

FDHs contain multiple sequence motifs; a series of amino acid residues integral for enzyme activity or structure, that are generally conserved throughout all FDHs. These are a dinucleotide-binding motif GxGxxG, part of the Rossman fold allowing for binding of flavin and NAD(P)H,^49^ and a tryptophan motif WxWxIP, suggested to block binding of substrate near the bound cofactor, thus preventing a monooxygenation reaction instead of halogenation.^37^ It is notable though, that GxGxxG, the flavin binding motif is present in almost all flavoenzymes. The WxWxIP motif or variants of this motif, is known to be absent in a number of FDHs including the more unusual Bmp5.^48^ A new motif, Fx.Px.Sx.G was recently reported. This, or variants of this motif in which G is replaced with a charge bearing residue, line the tunnel between the flavin binding site and the site of halogenation, may be found in all active FDHs.^47^ As such, it can be used as a method for quickly identifying candidate FDHs from uncurated sequence data. A complementary and overlapping motif FX[DE]PX[EFL] was subsequently identified by Lewis and coworkers.^157^

Notably, almost all halogenases studied, until recently, have been identified from bacteria and fungi, most being found due to their implication in the biosynthesis of a specific natural product or series of natural products. Iodination had also been missing from the FDH portfolio of reactions, though postulated to be involved in the biosynthesis of iodinated natural products.^50^ Whilst efforts had been made to explore the utilisation of enzymes such as RebH for iodination, incubation of such enzymes with iodide had been unfruitful, and competition studies had revealed that the presence of such salts procluded conversion, perhaps due to formation and inability to release the bulky product.^43^ More recently the FDH, PltM, was shown to be able to process sodium iodide, though preference for bromination, then chlorination was apparent.^51^ VirX1, the first halogenase to be characterised from a virus, is the first FDH for a preference for halogenation, and may be seen to have a large and readily accessible active site (Fig. 2A and B). Hydrophobic residues anchor the substrate in position, enabling regiospecific halogenation as guided by the proximal lysine (K79). Glutamate enables further activation, of otherwise less electrophilic substrates. It is postulated that by virtue of openness of this active site not only can a large range of small to sterically bulky substrates be processed, but halogenation by the largest and most readily oxidised of the halides is enabled.^47^

#### 1.2 Radical (X˙) halogenation

**Scheme 3:**
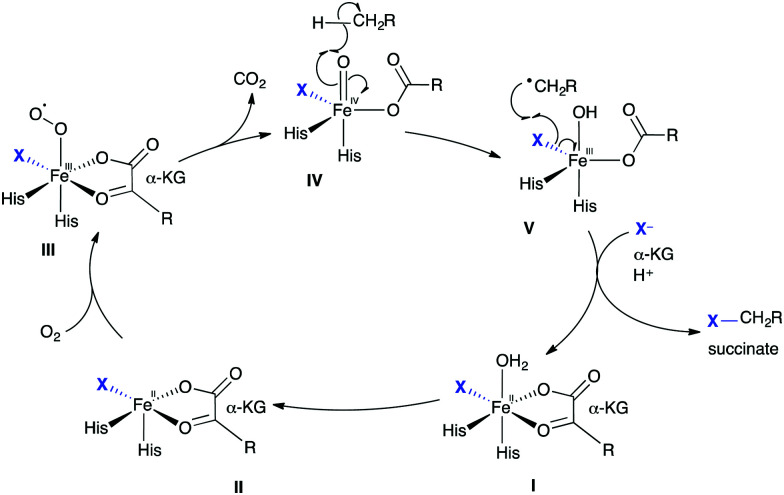
Radical halogenation. Biocatalytic cycle of a non-heme iron α-ketoglutarate-dependent halogenase, showing the release of halogenated product.

The halogenation of less electron-rich alkyl groups requires a different enzymatic strategy compared to the halogenation of activated aryl moieties.^52^ To achieve these energetically less favourable halogenations, radical chemistry is employed. Just as electrophilic FDH utilises similar enzymatic machinery to flavin dependent monooxygenases, analogously, non-heme iron α-ketoglutarate (KG)-dependent (NHFe) halogenases (NHFeHals) may well have evolved from their hydroxylase counterparts. Understanding of the catalytic cycle mediated by the NHFeHals is informed by the studies of the hydroxylases, which also require a non-heme iron centre, α-KG and O_2_ to function (Scheme 3). The catalytic cycle may notionally be considered to start with octahedral Fe^II^ coordinated in a bidentate fashion by α-KG, and a weakly bound water ligand that is subsequently replaced by molecular oxygen. The remaining three positions, a “facial triad,” in hydroxylases, consists of two histidine residues and the carboxylate of either an aspartate or glutamate residue at the active site.^53^ In halogenases, this carboxylate residue is usually found to be replaced by either an alanine or glycine, thereby enabling the halogen ion to coordinate at this vacant coordination site (Scheme 3, **I**). It has been shown that the weakly bound axial water ligand is initially displaced by substrate binding, triggering the subsequent reaction of **II** with molecular oxygen.^54^ Oxidative decarboxylation of **III** affords trigonal bipyramidal **IV**, with succinate coordinated, and is the driving force for the catalysis. This high valent, high energy Fe^IV^-oxido species is capable of abstracting H˙ from an otherwise unactivated alkyl group.^55^ The regio-selectivity of abstraction is governed by how the substrate is positioned within the enzyme's active site. Selectivity for reaction of generated substrate radical with Fe(iii)–X bond, leading to the desired halogenation over the Fe(iii)–OH bond, which would lead to hydroxylation, is thought to be controlled by careful substrate positioning over Fe^III^ species **V**.^56^ In HctB, a NHFeHal from hectochlorin biosynthesis, residues such as the Glu223 and Arg254 are thought to influence the charge density of the chlorine ligand, and are thus postulated to further promote halogenation.^57^ The resultant alkyl radical proceeds to extract X˙ from **V**, returning the metal centre to its Fe^II^ state.

Significant conformational changes are observed within these NHFe proteins. These changes are postulated to occur directly after oxygen binding and the subsequent decarboxylation event, orientating the substrate for the observed regioselective halogenation. Crystal structures of WelO5 with NO (an O_2_ analogue) reveal a significant change in conformation. Initially, NHFeHals appeared to be difficult to handle *in vitro* due to oxygen sensitivity of the Fe(ii) core,^58^ however, recent advances suggest an iron-reconstitution step can be performed to activate enzymes purified aerobically.^59^ The majority of NHFeHals, such as SyrB1,^58^ BarB1 and BarB2,^60^ and CurA halogenase,^61^ like the variant B FDHs, all required the substrate to be presented on carrier proteins, tethered *via* a phosphopantatheine linker (Ppant). In 2014, WelO5 was discovered, becoming the first member of a new family of NHFeHals (that we will refer to as variant A NHFeHals), which act on freestanding substrates.^62^ Excitingly, these open up the possibility for using such proteins more readily in biotransformations.

#### 1.3 Nucleophilic (X^−^) halogenation

Though the C–F bonds are prevalent in pharmaceuticals, and fluorine is the most abundant of the halogens on earth, few fluoro-metabolites are known. So far, 13 fluorometabolites, 7 of these being closely related fluorinated lipids, have been discovered. The bacterium *Streptomyces cattleya* is known to biosynthesise three of this limited portfolio of fluorinated metabolites: fluoroacetate, 4-fluorothreonine, and (2*R*,3*S*,4*S*)-trihydroxy-5-fluoropentanoic acid. These compounds are generated by the “fluorinase,” more formally named adenosyl fluoride synthase or FlA, that was discovered by O’Hagan and co-workers.^63^ FlA is closely related to the more recently discovered SalL, an enzyme that mediates chlorination in salinosporamide biosynthesis.^64^ These enzymes are members of a rare group of nucleophilic halogenases, all of which utilise *S*-adenosyl-l-methionine **23** (SAM) as substrate. *Streptomyces calvus* is capable of biosynthesising a fourth bacterial fluorometabolite, nucleocidin.^65^ Intriguingly, a gene with similarity to that which encodes the fluorinase is absent from the genome of this organism implying that a further group of halogenases capable of mediating C–F bond formation exists. The dearth of fluorometabolites found in nature may perhaps be attributed to a number of factors. With the high electronegativity of fluorine, the possibility of oxidation to generate a fluorous electrophile or even a fluorous radical is energetically challenging, and no enzymes have so far shown this capability. Also, fluoride, though abundant, is generally present in minerals such as fluorospar (fluorite), which have high lattice energies and poor solubilities. Once in solution, fluoride is surrounded by a sheath of water, its high enthalpy of hydration (490 kJ mol^−1^) precludes ready displacement of the water molecules to access the naked fluoride ion required for nucleophilic attack.^66^

**Scheme 4:**
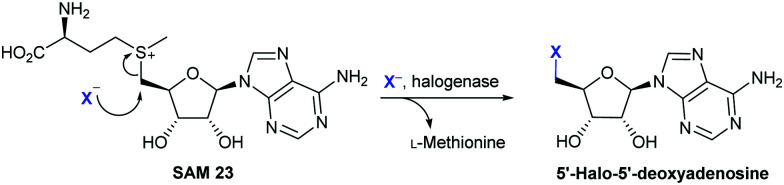
Nucleophilic halogenation. Halogenation of *S*-adenosyl-l-methionine **23** (SAM) by X^−^ mediated by a nucleophilic halogenase, yielding l-methionine and 5′-halo-5′-deoxyadenosine.^67^

The nucleophilic halogenases characterised so far all operate by holding the halide proximal to the electrophile, SAM. Crystallographic studies of the fluorinase reveal a small pocket, the fluoride binding site, giving insight into how displacement of the hydration sphere might be achieved. Whilst predominantly hydrophobic, this small pocket contains a serine.^67^ Dehydration is proposed to occur when SAM binds with the positive charge on the sulphur possibly assisting with stabilisation of the naked fluoride. The requirement for the fluoride ion to bind first in order for a productive complex to be formed, is argued based on observed substrate inhibition by SAM. Within the crystal structure, SAM may be seen bound with its ribose ring forced into a high energy, eclipsed conformation. The halide anion is positioned collinearly with the SAM C–S bond, thus promoting an S_N_2 backside attack (Scheme 4).^67^ When the enzyme is crystallised in the presence of both SAM and fluoride, the products l-methionine and 5′-fluoro-5′-deoxyadenosine (5′-FDA) with the ribose ring in the relaxed conformation may be observed.

The fluorinase has a strong preference for fluoride, but can incorporate chloride to a low level.^68^ SalL, from *Salinispora tropica* bacterium has 54% sequence similarity to the fluorinase, and in the halide binding pocket, a glycine residue resides in place of a serine. This enzyme shows a strong preference for chlorination and, whilst capable of utilising bromide and iodide, it cannot process fluoride.^64^

Notable uses of the fluorinase include exploration of its application to ^18^F incorporation for positron emission tomography (PET),^69^ and it's the utilisation of the fluorinase in parallel with a polyketide synthase to enable synthetic biological access to simple fluorinated polyketides.^70^

#### 1.4 Biocatalytic potential

The halogenases offer great potential for use as biocatalysts. To realise the application of these important enzymes, the regio-control, -selectivity and reaction rate afforded by the different classes of enzymes is important to consider. The reported exploration of kinetics for the halogenases in the literature is both sparse and varied, and can be challenging to compare due to the different ways in which the data are obtained. We compile a representative overview of the kinetic analyses available for different families of halogenases for a brief comparison between families (Table 1), as well as a glossary of the halogenases that have been structurally characterised so far (Table 2). Haloperoxidases, such as CPO, may be seen to have high turn-over numbers (*K*_cat_ 33 ± 1 min^−1^), yet such catalysts that may be utilised to halogenate a broad series of substrates (see Section 2) lack regio-control. Conversely, the NHFeHals also have a high turn-over number, mediate a highly regio-selective reaction, but the natural reaction that its catalyses is much slower than CPO (*K*_cat_/*K*_m_ 0.0525 *vs.* 1.22 μM^−1^ min^−1^, Table 1).

**Table 1:** Representative electrophilic (VirX1, PltM, PyrH, CPO, V-BrHPO), nucleophilic (FlA) and radical halogenases (WelO5, PfHalA) and their kinetic parameters with respect to substrate at set concentrations of other cofactors. ND = no data published

Enzyme	Substrate	Classification	Halide	*k*_cat_ (min^−1^)	*K*_M_ (μM)	*k*_cat_/*K*_M_ (μM^−1^ min^−1^)	Ref.
VirX1	6-Azaindole	FDH	I	5 ± 0.5	28 ± 2	0.179 ± 0.004	47
			Br	2.4 ± 0.6	53 ± 3	0.044 ± 0.008	
PltM	Resorcinol	FDH	Br	1.9 ± 0.1	0.71 ± 0.3	2.7 ± 0.6	51
			Cl	2.3 ± 0.1	0.076 ± 0.013	30 ± 5	
PyrH	Tryptophan	FDH	Cl	3.56 ± 1.1	109 ± 44	0.0325 ± 0.017	51
KtzQ	7-Cl-l-Tryptophan	FDH	Cl	1.4	114	0.012	82
MalA	Premalbrancheamide	FDH	Cl	0.08 ± 0.05	7.0 ± 2.9	11.49 ± 0.02	46
WelO5	12-Epi-fisherindole U	NHFeHal	Cl	1.8 ± 0.2	ND	ND	83
PfHalA	Lysine	NHFeHal	Cl	14.7 ± 0.71	280 ± 60	0.0525 ± 0.0118	84
AdeV	2′-Deoxyadenosine monophosphate	NHFeHal	Cl	0.6	2500	0.24 × 10^−3^	85
CPO	Catechol	HPO	Cl	33 ± 1	27 ± 8	1.22 ± 0.36	86
V-BrHPO	2-Chlorodimedone	V-HPO	Br	5	ND	ND	26
FlA	SAM	Nucleophilic	F	0.07 ± 0.001	6.5 ± 0.3	0.0108 ± 0.0005	87

Glossary of structurally characterised halogenases, arranged by class

**Table:** 

Enzymes	No. residues	PDB code	Cofactors and ligands	Identification time	Relevant BGC	Oligomer	Reported substrates	Halogen	Halogenation stage	Source	Subgroup	Ref.
PrnA	538	2AR8	FAD, Cl^−^, 7-Cl-Trp	2005	Pyrrolnitrin	Dimer	Tryptophan 7C, indolic substrates	Cl, Br	Early stage	*Pseudomonas fluorescens*	A	37
		2APG	FAD, Cl^−^,									37
		2AQJ	FAD, Cl^−^, Trp									37
		2ARD	FDA									37
		4Z43 (E450K)	FAD, Cl^−^	2015								79
		4Z44 (F454K)	FAD, Cl^−^, PO4									79
		2JKC (E346D)	FAD, Cl^−^, Trp	2008								39
												
RebH	530	2OA1	FAD, ADN, Trp, Cl^−^	2008	Rebeccamycin	Dimer	Tryptophan 7C, indolic substrates, aniline substrates	Cl, Br	Early stage	*Lechevalieria aerocolonigenes*	A	88
		2O9Z	PO4									88
		2OAL	FAD, Cl^−^	2007								43
		2OAM	Apo form									43
		2E4G	Trp									43
		4LU6 (thermostabilized)	PO4	2013								89
		6P00 (11 mutations)	FAD	2020								TBP
		6P2V (6 mutations)	FAD									TBP
		7JU0 (1 mutation)	FAD, TSS									TBP
												
CndH^a^	512	3E1T	FAD, Cl^−^	2009	Chondrochloren	Monomer	Aliphatic substrates	Cl	Early stage	*Chondromyces crocatus*	B	36
												
PyrH	511	2WET	FAD, Trp, Cl^−^	2010	Pyrroindomycin	Dimer	Tryptophan 5C	Cl, Br	Early stage	*Streptomyces rugosporus*	A	90
		2WEU	Trp	2009								90
		2WES (E46Q)	FAD, Cl^−^	2009								90
												
CmlS^a^	570	3I3L	FAD	2010	Chloramphenicol	Monomer	Aliphatic substrates	Cl, Br	Late stage	*Streptomyces venezuelae*	A	44
												
PltA	455	5DBJ	FAD, Cl^−^	2015	Pyoluteorin	Dimer	Pyrrolic substrates	Cl	Early stage	*Pseudomonas fluorescens*	B	91
												
Mpy16	447	5BUK	FAD	2016	Pyrrolyl-*S*-Mpy15	Monomer	Pyrrolic substrates	Cl	Late stage	*Streptomyces* sp. *CNQ-418*	B	48
												
Th-Hal	510	5LV9	—	2016	—	Monomer	Tryptophan	Cl, Br	—	*Streptomyces violaceusniger*	A	92
		5LVA	FMN	2016								92
SttH	519	5HY5	FAD, Cl^−^	2016	—	Dimer	Tryptophan 6C, *N*-methyltryptophan, kynurenine, anthranilamide	Cl, Br	—	*Streptomyces toxytricini*	A	80
												
Bmp2	409	5BVA	FAD, EDO	2016	Tetrabromo-pyrrole	Monomer	Pyrrolic substrates	Br, I	—	*Pseudoalteromonas luteoviolacea*	B	48
		5BUL (Y302S F306V A345W)	FAD									48
												
MalA	667	5WGZ	FAD, IM7, Cl^−^	2017	Malbrancheamide	Monomer	Premalbrancheamide, malbrancheamide B, isomalbrancheamide B	Cl, Br	Late stage	*Malbranchea aurantiaca*	A	46
		5WGV (C112S C128S)	FAD, PM7, Cl^−^									46
		5WGU (E494D)	FAD, PM7, Cl^−^									46
		5WGX (H253A)	FAD, MB5, Cl^−^									46
		5WGY (C112S C128S)	FAD, MB5, Cl^−^									46
		5WGR	FAD, PM7, Cl^−^									46
		5WGS (H253F)	FAD, PM7, Cl^−^									46
		5WGT	FAD, PM7, Cl^−^									46
		5WGW	FAD, MB5, Cl^−^									46
												
BrvH	502	6FRL	—	2018	—	Dimer	Indolic substrates	Br > Cl	—	*Brevundimonas BAL3*	B	93
												
Tar14	532	6NSD	FAD	2019	Taromycin	Dimer	Tryptophan	Cl, Br	Early stage	*Saccharomonospora* sp. *CNQ-490*	A	94
												
Thal	534	6H43	PO4	2019	Thienodolin	Dimer	Tryptophan 6C, indolic substrates	Cl, Br	Early stage	*Streptomyces albogriseolus*	A	81
		6H44	Trp, PO4	2019								81
		6IB5 (Thal-RebH5, 5 mutations)	PO4	2019								81
		6SLT	FAD, AMP, Trp, PO4	2019								95
		6SLS	FAD, PO4									95
		7AQV (N-6His-Thal-RebH5, 5 mutations)	—	2020								TBP
		7AQU (N-6His-Thal-RebH5, 5 mutations)	BCN, SER, ALA, GLY	2020								TBP
		7CU0	Trp	2020								96
		7CU1	FAD, AMP	2020								96
		7CU2	FDA	2020								96
												
PltM	522	6BZN	—	2019	Pyoluteorin	Monomer	Phenolic substrates, aniline substrates	Cl, Br, I	—	*Pseudomonas protegens Pf-5*	A	51
		6BZZ	FAD	2019								51
		6BZA	FAD, 13X, Cl^−^	2019								51
		6BZQ	FAD, Br^−^, Cl^−^	2019								51
		6BZT (L111Y)	FAD, Br^−^, Cl^−^	2019								51
		6BZI	EMC, HG	2019								51
												
Virx1	531	6QGM	Apo form	2019	—	Trimer	Aromatic substrates, heterocycles, azaspirocycles	I > Br > Cl	—	*Cyanophage Syn10*	A	47

a No *in vitro* activity demonstrated to date.

b Natural protein length from UniProt.

c Not specifically mentioned, tested by standard substrate monochlorodimedone/TB iodoperoxidase assay.

d FlA is not in a BGC, but participates in the synthesis of fluoroacetate and 4-fluorothreonine.

**Table:** 

Classification	Enzymes	No. residues^b^	PDB code	Cofactors and ligands	Idt. time	Relevant BGC	Oligomer	Reported substrates	Halogen	Source	Ref.
Vanadium-dependent haloperoxidases	Ci-VCPO	609	1VNC	VO4, AZI	1996	—	Monomer	Chlorinated 1-(4-ethoxy-3-methoxyphenyl)-2-(2-methoxyphenoxy)-1,3-dihydroxypropane	Cl, Br	*Curvularia inaequalis*	97
			1VNS	SO4	1999						98
			1VNI	Apo form							98
			1VNH (H496A)	VO4							98
			1VNG (H404A)	VO4							98
			1VNF (R360A)	VO4		—					98
			1VNE (D292A)	VO4							98
			1IDQ	VO4	1997	—					99
			1IDU	VO4		—					99
			3BB0	PO3	2008						100
										
	An-VBPO1	556	1QI9	VO4, I^−^	1999	—	Dimer	2-Methylindole, 2-phenylindole, phenolsulfonephthalein, derivatives of *O*-methyl pyrrole-2-carboxylate	Br, I	*Ascophyllum nodosum*	101–103
										
	An-VBPO2	597	5AA6	VO4	2015	—	Hexamer	Refer to An-VBPO1	Br, I	*Ascophyllum nodosum*	TBP
										
	Co-VBPO	596	1QHB	PO4	1999	—	Dodecamer	(*E*)-(+)-Nerolidol	Br	*Corallina officinalis*	104
	Cp-VBPO	598	1UP8	PO4	2005	—	Dodecamer	c	Br	*Corallina pilulifera*	105
										
	NapH1	531	3W35 (Apo form)	—	2012	Napyradiomycin	Dimer	Polyketide-terpenoid substrates	Cl, Br	*Streptomyces* sp. *CNQ525*	TBP
			3W36	VO4	2012						TBP
										
	Zg-VIPO1	458	4CIT	VO4	2014	—	Monomer	TB iodoperoxidase assay	I	*Zobellia galactanivorans*	106
										
	Zg-VIPO2	458	4USZ	VO4	2014	—	Monomer	TB iodoperoxidase assay	I	*Zobellia galactanivorans*	106
										
	AmVHPO	639	5LPC	PO4	2016	—	Dodecamer	Aromatic substrates	Cl, Br	*Acaryochloris marina*	107
											
Heme-dependent haloperoxidases	HI-HPO	373	1CPO	HEM	1995	Caldariomycin	Monomer	1,3-Cyclopentanedione, electron-rich aromatic substrates	Cl, Br	*Leptoxyphium fumago*	108
			2CPO	HEM							108
			2CJ0	HEM	2006						109
			2CIW	HEM, I^−^							109
			2CIV	HEM, Br^−^							109
			2CJ2	HEM, FMT							109
			2CJ1	HEM, FMT							109
			2CIZ	HEM, Br^−^							109
			2CIY	HEM, Br^−^, CYN, DMSO							109
			2CIX	HEM, CEJ, Br^−^, EDO							109
NHFe dependent halogenases (NHFeHal)	CytC3	319	3GJA	ACT	2009	γ,γ-Dichloroaminobutyrate	Monomer	l-2-Aminobutyric acid	Cl	*Streptomyces* sp.	110
			3GJB	AKG, ACT, Fe^2+^							110
										
	SyrB2	310	2FCT	DSU, AKG, Cl^−^, Fe^2+^	2006	Syringomycin E	Monomer	l-Threonine	Cl, Br	*Pseudomonas syringae*	111
			2FCV	DSU, AKG, Br^−^, Fe^2+^							111
			2FCU	DSU, AKG							111
										
	CurA-Hal	2311	2LIU	—	2011	Curacin A	Domain (NMR structure)	3-Hydroxyl-3-methyl-glutaryl	Cl	*Lyngbya majuscula*	111
			2LIW	PNS, MAH	2011		Domain (NMR structure)				111
										
	WelO5	315	5IQS	AKG, Fe^2+^, Cl^−^	2016	Welwitindoline	Monomer	12-Epi-fischerindole U	Cl, Br	*Hapalosiphon welwitschii*	111
			5IQT	6CU, AKG, Fe^2+^, Cl^−^							111
			5IQU (G166D)	6CU, AKG, Fe^2+^							111
			5IQV	6CU, AKG, Fe^2+^, Cl^−^, NO							111
			5J4R	AKG, Ni^2+^	2016						TBP
			5T22	AKG, Ni^2+^	2016						TBP
			5TRQ	SIN, ACT, Ni^2+^	2016						TBP
SAM (*S*-adenosyl-l-methionine)-dependent halogenases	FIA	299	1RQP	SAM	2004	d	Hexamer	SAM	F	*Streptomyces cattleya*	67
			1RQR	5FD, MET							67
			2C2W	5CD, Cl^−^	2005						68
			2C4T	SA8, Cl^−^	2007						TBP
			2C4U	—	2006						112
			2C5B	5F1, MET							112
			2CBX	CC5							112
			2CC2	—							112
			2C5H	3D1, MET, Cl^−^	2006						TBP
			2V7T (S158G)	SAH, Cl^−^	2008						87
			2V7U (S158G)	SAM, Cl^−^							87
			2V7V	5FD							87
			2V7W (S158G)	5FD							87
			2V7X (S158G)	5FD, MET							87
			4CQJ	EFA	2014						TBP
			5FIU	Y3J, TLA	2015						113
			5LMZ	1DA, Cl^−^	2016						114

### 2. Natural and unnatural aromatic substrate scope for biocatalytic halogenation

#### 2.1 Indolic substrates

##### 2.1.1 Early stage halogenation in biosynthetic pathways

Indoles are commonly utilised motifs in medicine and agrochemistry, and new tools that enable their chemical diversification are desirable. There are a series of FDHs that allow for this diversification, *via* halogenation. Many FDHs mediating the regiospecific halogenation of the indole moiety of tryptophan have been found so far. Most of these enzymes have been found to be involved in the early stages of bioactive natural product synthesis. Such enzymes include PrnA and RebH, tryptophan 7-halogenases, from the first step of the biosynthesis of the antibiotic pyrrolnitrin **24** and the indolocarbazole anticancer agent rebeccamycin **36**, respectively.^35,43^ Tryptophan 6-halogenases include ThdH (alternatively known as ThaI) from the first step of thienodolin **25** biosynthesis, a plant growth promoting compound,^71^ SttH identified *in silico* adjacent to an NRPS cluster,^72^ SatH from *Streptomyces albus*,^73^ BorH, a thermophilic halogenase from borregomycin **33–35** biosynthesis,^74^ and AfnX responsible for generating the 6-chlorotryptophan precursor of alboflavusin A **26**. Alboflavusin is an analogue of the dimeric non-ribosomal peptide (NRP) anti-tumor agent and apoptosis inducing agents, himastatin and chloptosin.^75^

Though less common so far, 5-chlorotryptophans may also be seen as motifs in natural products. Examples include the antibiotic pyrroindomycin B **27**; the first step in the biosynthesis of this compound is the generation of 5-chlorotryptophan by PyrH.^76^ Sequence analysis of *Xenorhabdus szentirmaii* revealed a further flavin dependent tryptophan 5-halogenase XszenFHal. Though the natural function of this halogenase remains unknown, it was demonstrated to be capable of regioselectively halogenating a series of indolic and anthranilic substrates.^77^

The first successful demonstration of the ability to modify the regioselectivity of a FDH was *via* site directed mutagenesis of tryptophan 7-halogenase PrnA. The modification of a large phenylalanine residue in the active site to a smaller alanine allowed for a different binding orientation of tryptophan, leading to halogenation at the 5 position, though it must be noted that halogenation at the 7 position still dominated (2 : 1 ratio of 7-/5-bromination).^78^ Despite useful changes in selectivity being rare, there are a number of further successes.^42,79^ Positioning of the lysine residue over the substrate was thought to be critical to site of halogenation. Various studies toward gaining deeper understanding of subtle factors beyond the positioning of the lysine residue in the active site have been carried out, including structural comparison of PyrH, SttH and PrnA, and, specifically, a loop proximal to the active site, informing the design of mutants with modified regiochemistries.^80^ Modifications to ThaI, to promote chlorination at the 7 position, rather than the 6 position have also been reported.^81^

Biosynthesis of 4-chlorotryptophan remains a mystery. Although it was isolated from immature pea seedlings in 1970, and postulated to be a precursor to a plant growth hormone,^115^ the halogenase has not yet been found, hinting that it may be strikingly different to the other, well known FDHs. There have also been fewer reports of natural products containing 2-halotryptophans, perhaps due to their inherent instability. The keramamides **30**^116^ and linked family of jaspamides, chondramides **28** are two exceptions, with CmdE implicated in the generation of 2-chlorotryptophan,^117^ though for this enzyme, no activity with free tryptophan can be shown. It is not clear as to whether lack of observation of production of this species by CmdE is due to the enzyme requiring a different substrate or due to the instability of the product. 2-Chlorotryptophans are very unstable and both the free amine and carboxy groups might be considered to promote dechlorination.

**Fig. 3:**
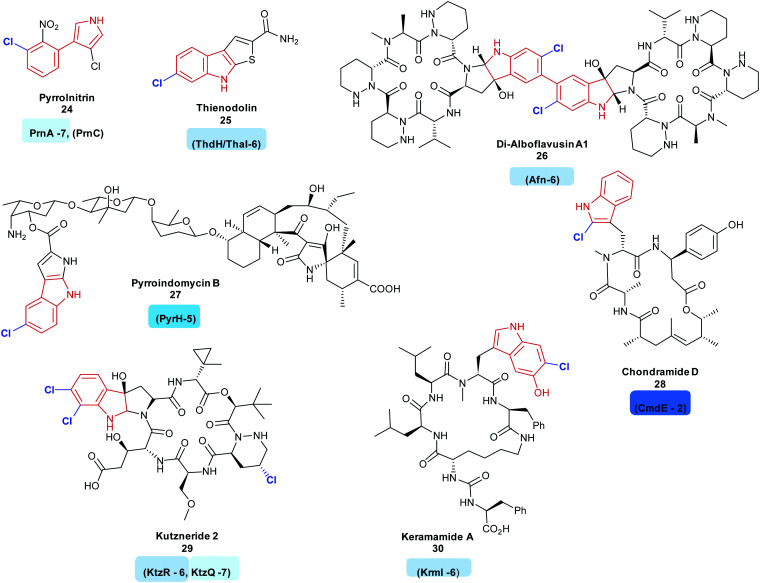
Halogenated natural products arising from the halogenation of indolic substrates: all include regioselective halogenation of tryptophan as a first step in their biosynthesis (tryptophan halogenase name and regiochemistry noted below each corresponding natural product). Examples include (C7) pyrrolnitrin **24**,^120^ (C6) thienodolin **25**,^71^ Di-alboflavusin A1 **26**,^75^ (C5) pyrroindomycin B **27**,^76^ (C2) chondramide D **28**^117^ (C6, C7) kutzneride **29**,^121^ keramamide A **30**^116^ Tryptophan halogenases involved in the biosyntheses of these natural products are depicted, and are colour coded in accordance with the regiochemistry of the halogenation that they mediate. Chlorination is indicated in blue. Different regio-chemistries for chlorination are represented by different shades of blue.

Tryptophan halogenases capable of halogenating substituted tryptophans are also known. These include the tryptophan 6-halogenase KtzR, which acts in tandem with KtzQ in the first steps of kutzneride **29** assembly, installing a second chlorine into the 7-chlorotryptophan that KtzQ generates^118^ and KrmI, another tryptophan-6-halogenase, which processes 4-hydroxy-tryptophan as its natural substrate, though the wild type enzyme shows ability to process a fairly broad range of other substrates. KrmI is an unusual halogenase, involved in the biosynthesis of the keramamides **30**, this FDH is translationally fused to a ThiF protein^119^ (Fig. 3).

**Fig. 4:**
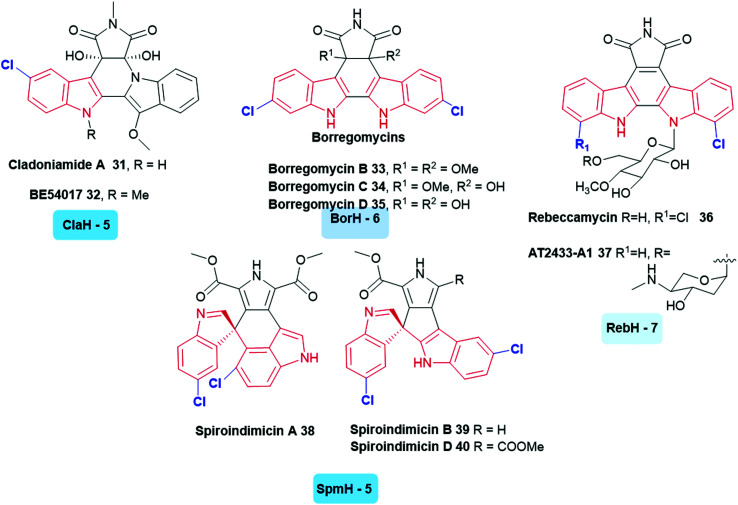
Naturally occurring halogenated bisindole alkaloids. These compounds incorporate a halogen into tryptophan in the first stage of their biosynthesis. (The tryptophan halogenase responsible and its regioselectivity that it confers, are noted below each natural product.) Examples include indolocarbazoles (C5) cladoniamide A **31**,^122^ (C5) BE-54017 **32**,^128^ (C6) borregomycins B **33**, C **34** and D **35**^123^ (C7) rebeccamycin **36**,^40^ (C7) AT2433-A1 **37**,^129^ (C5) spiroindimicins **38–40**.^125^ Non-halogenated analogues of the latter have been accessed through deactivation of SpmH.^125^ Tryptophan halogenases involved in the biosyntheses of these natural products are depicted, and are colour coded in accordance with the regiochemistry of the halogenation that they mediate. Chlorination is indicated in blue. Different regio-chemistries for chlorination are represented by different shades of blue.

A large variety of symmetrically and asymmetrically halogenated indolocarbazole compounds are known, including the cladoniamides **31**, **32**,^122^ borregomycins **33–35**,^123^ and rebeccamycins **36**, **37**.^40^ From genomic analysis of the producing organisms, the first step in the biosynthesis of these compounds also involved the generation of 5, 6 or 7-halotryptophan. This biosynthetic logic has been utilised to enable the generation of combinatorial libraries of known and novel compounds in which halogenation and glycosylation patterns are modulated.^124^ Similar to these compounds are the bisindole alkaloids indimicins and related spiroindimicins **38–40** (Fig. 4).^125^

**Fig. 5:**
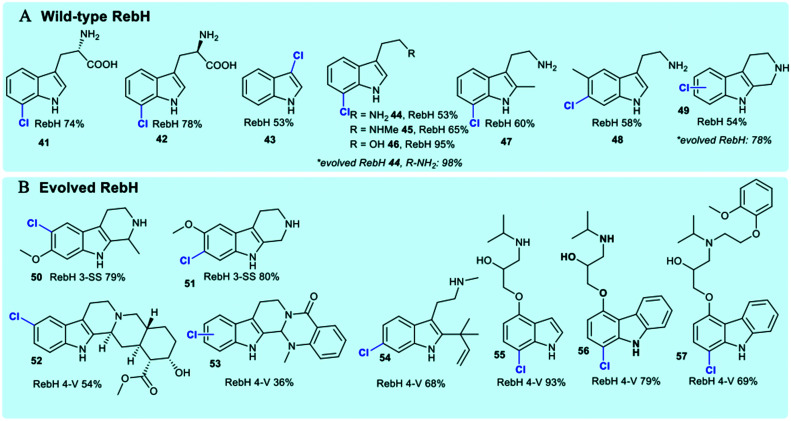
Representation of some of the diverse substrate scope of tryptophan halogenases. Enzymes include (A) wild-type RebH chlorinating indoles;^42,126^ (B) evolved RebH 3-SS and RebH 4-V which accept tricyclic tryptoline and large indole carbazoles.^127^

The tryptophan halogenases have been previously thought to possess only modest innate substrate flexibility. RebH is capable of processing tryptolines, whilst KrmI will halogenate unsubstituted indoles and fluoro-tryptophan.^42,119^ The tryptophan halogenases continue to be subject to extensive rational reengineering and directed evolution due to their potential as tools for biocatalysis. In addition to studies that explore and modulate factors governing regiocontrol, considerable effort continues to be invested in extending substrate scope.^41^ Wild-type RebH (the tryptophan 7-halogenase from rebeccamycin **36** biosynthesis) has been demonstrated to be capable of regioselectively halogenating a series of indolic (Fig. 5) and arene substrates (Fig. 9).^126^

Natural substrate scope for the FDHs RebH, GsfI, ThaI and Rdc2 is seen to be a little broader, and these enzymes have capability to process a range of indolic substrates.^42^ Directed evolution of RebH has been employed by the Lewis group to tune the enzyme for use in 7- **44**, 6- and 5-chlototryptamine generation enabling quantitative conversions in some instances, and high selectivities.^42^ Evolved RebH variants have been employed, achieving selective late stage functionalisation of chlorination of sterically bulky substrates, including tricyclic tryptolines **50–53** and large indole carbazoles **56**, **57**, improving conversions of these unnatural substrates as well considerably extending substrate scope from what was previously known (Fig. 4).^126,127^

Production of halogenated indolic substrates by flavin-dependent halogenases is commonly reported at small scale.^47,126,127^ The use of Cross-Linked Enzyme Aggregates (CLEAs), in which the physical aggregation of enzymes with a cross-linking agent is mediated, can enable enzyme stabilisation and enhanced yields. By crosslinking RebH and PrnF (the PrnA-related reductase) with glutaraldehyde, Sewald was able to achieve yields on a gram scale with CLEAs for the halogenation of l-tryptophan, d-tryptophan and l-5-hydroxytryptophan, establishing the foundations for halogenase biocatalysis on a preparative scale^130^ (see Section 3).

#### 2.2 Phenolic substrates

**Scheme 5:**
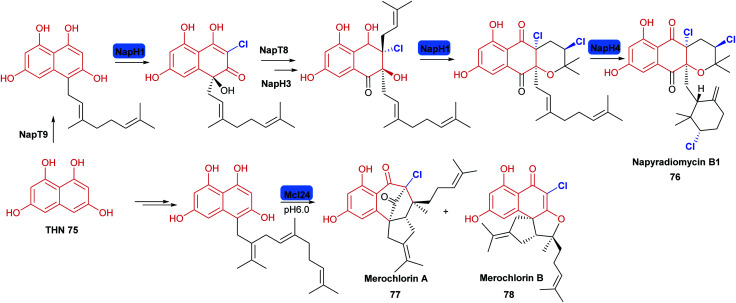
Halogenation in the biosynthesis of the phenolic napyradiomycin **76** and merochlorins **77, 78**: these natural products are synthesised from polyketide derived tetrahydroxynaphthalene (THN), the biosynthesis of these suites of metabolites involve versatile VHPOs and employ a chemical logic of chloronium formation promoting enantioselective intramolecular cyclisation.^153,155^ Chlorination is indicated in blue.

Phenolic quinolines attract attention for their many and varied medicinal properties such as antimalarial, antibacterial and anticancer activities.^131^ Structure–activity relationship (SAR) reveal that installation of a chlorine or bromine atom within the quinoline ring of antitubercular quinolines can enhance their activity.^131^ Beyond antitubercular uses, chlorine-containing 8-hydroxyquinoline derivatives are being explored for treatment of Alzheimer's disease.^132^ Phenols, being electron-rich, are frequently seen to be good substrates for electrophilic enzymatic halogenation (Fig. 8 and Scheme 5). While the haloperoxidases generally confer limited specificity, the FDHs show good levels of regiocontrol for accessing such halogenated compounds.

##### 2.2.1 Early stage halogenation by FDH variant B enzymes

**Fig. 6:**
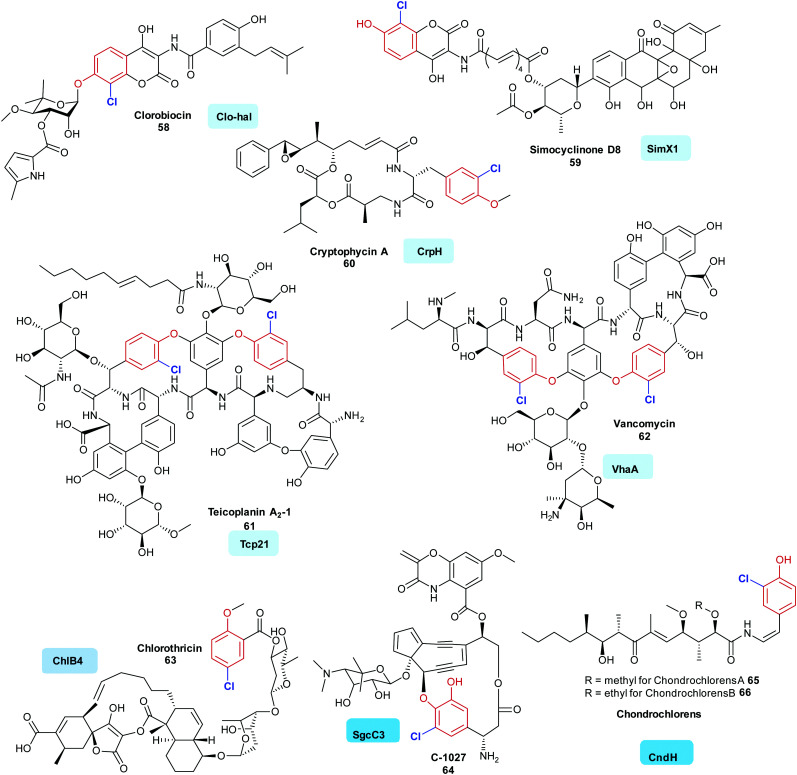
Representative bioactive natural products containing halophenol motifs, where the halogen is installed by a variant B FDH. Clorobiocin **58**,^133^ simocyclinone D8 **59**,^134^ cryptophycin A **60**,^135^ are all postulated to be generated from the halogenation of an enzyme tethered tyrosine residue, in the first step (halogen added *ortho* in EAS fashion as mediated by Clo-hal, SimX1 and CrpH respectively). *In vitro* evidence supporting the early stage halogenation of an enzyme tethered tyrosine exists for teicoplanin **61** and vancomycin **62** (mediated by Tcp21^136^ and VhaA).^136^ C-1027 **64**^137^ and the chondroclorens **65**, **66** are both postulated to arise from the halogenation of an enzyme tethered analogue of tyrosine, in the first stages of the biosynthesis,^36,138^ whereas a first step of chlorothricin **63** assembly is thought to be the halogenation of enzyme tethered salicylate (catalysed by ChlB4).^139^ Chlorination is indicated in blue. Different regio-chemistries for chlorination are represented by different shades of blue.

Whilst there has been considerable and detailed investigations of a number of the variant A halogenases that work on substrates that are not covalently tethered to a carrier enzyme (in particular the tryptophan halogenases), fewer *in vitro* investigations of variant B halogenases have been published. However, a diverse range of bioactive natural products that contain halogenated phenols have been isolated. Here, the operation of variant B FDHs may be seen to be prevalent, and investigations indeed indicate many of the substrates to be enzyme tethered. Halophenol-containing natural products include the actinobacteria-produced aminocoumarins clorobiocin **58** and simocyclinone D8 **59**. The initial steps of the biosyntheses of these metabolites are postulated to include halogenation of enzyme-tethered tyrosine (mediated by Clo-hal and SimX1, respectively).^133,134^ Evidence points to a similar halogenation strategy also being utilised in the biosynthesis of the peptidic cyanobacterial products aeruginosin (AerJ), cyanopeptolin (McnD), and cryptophycin A (CrpH) **60** of mixed PKS NRPS origin.^135,140^ A similar biosynthetic logic is also predicted for the glycopeptide antibiotics (GPAs). Vancomycin **62** (VhaA) and teicoplanin **61** (Tcp21), are two potent GPAs; again, these compounds contain chlorinated tyrosine residues. In the case of the GPA teicoplanin **61**, careful *in vitro* investigations have shown that Tcp21 accepts the peptidyl carrier protein (PCP) supported aminoacyl substrate (tethered tyrosine), but will not accept the dipeptide or extended peptides as substrate.^136^ In teicoplanin biosynthesis, this amino acid residue is likely to be β hydroxylated at a later stage by a non-heme iron oxygenase, whilst in balhimycin biosynthesis OH-Tyr, and not tyrosine, is incorporated by the NRPS machinery.^141^ Analogues of tyrosine may also be accepted by halogenases. SgcC3 accepts β-tyrosine, probably also in carrier protein tethered form, in the biosynthesis of the enediyne C-1027 **64**.^137^ In a similar manner, enzyme tethered 3-hydroxy-4-methoxy-l-phenylalanine is seen to be processed to 2-chloro-3-hydroxy-4-methoxy-l-phenylalanine by Pep1 in the first steps of the biosynthesis of pepticinnamin G.^142^ In chondrochloren **65**, **66** biosynthesis, CndH is indicated to process a different, perhaps decarboxylated yet enzyme tethered analogue of tyrosine.^36^ Whilst many of the halogenases identified so far are seen to accept electron-rich aromatic amino acids in the biosynthesis of chlorothricin **63**, ChlB4 is postulated to act on the small polyketide subunit, 6-methyl salicylic acid^139^ (Fig. 6).

##### 2.2.2 Late stage halogenation by variant A FDHs

**Fig. 7:**
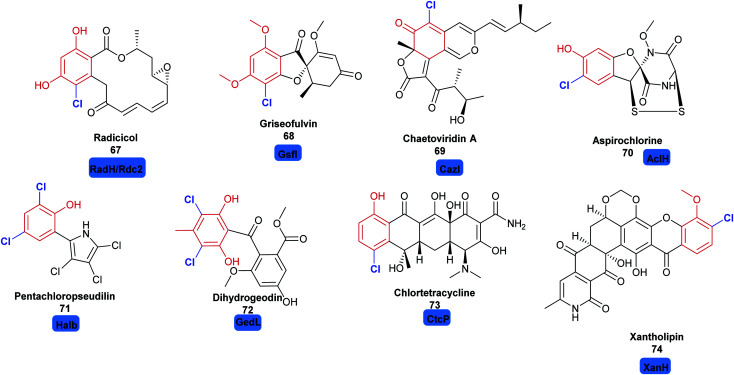
Halogenated natural products where installation of halogen on a structurally complex non-tethered freestanding phenol is performed selectively by a halogenase: including radicicol **67**,^143^ griseofulvin **68**,^145^ chaetoviridin A **69**,^146^ aspirochlorine **70**,^149^ pentachloropseudilin **71**^151^ dihydrogeodin **72**,^148^ chlortetracycline **73**^147^ and xantholipin **74**.^150^ Chlorination is indicated in blue.

Whilst the variant B FDHs are not easily amenable to *in vitro* investigations, and are indicated to have a very limited substrate scope, the variant A FDHs are less limited by these problems and offer great opportunity for harnessing as biocatalysts. Amongst the most exciting of these, are enzymes that are reported to act at a late stage of the biosynthesis of structurally complex and sterically bulky phenolic/quinolic natural products. Rdc2^143^ and RadH^144^ share 87% sequence similarity; these two enzymes are identified to act in the final steps of biosynthesis of the fungal polyketide natural product radicicol **67**, an Hsp90 inhibitor, within the fungi *Pochonia chlamydosporia* and *Floropilus chiversii* respectively. GsfI operates in the final stages of the assembly of griseofulvin **68**, a clinically utilised antifungal agent (Fig. 7).^145^ TiaM from tiacumicin B biosynthesis operates in the final step of the biosynthetic pathway, functionalising a complex and glycosylated polyketide generating both the mono and dihalogenated products.^226^

CazI, from chaetoviridin A **68** biosynthesis, is postulated to operate on a complex phenolic intermediate, in the final stages of this cytotoxic polyketide.^146^ In a similar manner, CtcP is seen to halogenate an advanced type II, aromatic polyketides core, in the biosynthesis of chlortetracycline **73**.^147^ Heterologous pathway reconstruction studies indicate that in geodin biosynthesis GedL dichlorinates the advanced PKS metabolite sulochrin, directly affording dihydrogeodin **72**.^148^ Aspirochlorine **70** is an epidithiodiketepiperazine (ETP) toxin, produced by *Aspergullus orizae*, a mold used for millennia in Asian cuisine. The first stages of the biosynthesis of aspirochlorine involve the incorporation of two phenylalanines into a symmetric diketopiperazine (DKP). Intriguingly, one phenylalanine undergoes a C–C cleavage to convert it into glycine. The other phenylalanine is hydroxylated. AclH, operates in the final step of the biosynthesis, chlorinating the hydroxylated phenylalanine residue and affording the toxin.^149^ XanH, a bifunctional FDH fused to its concomitant flavin reductase, has been shown capable of regioselectively chlorinating a complex late stage xanthone intermediate on the path to xantholipin **74**.^150^ Pentachloropseudilin **71** has a series of interesting biological activities including inhibiting TGF-beta signalling, and impairing angiogenesis; yet its exact biosynthesis remains unclear. HalB was found in a cosmid library of the producing organism; the first halogenase identified from an actinomycetes. Notably, HalB shows 55% sequence identity to the pyrrole FDH PrnC. HalB has been shown to be capable, *in vitro*, of installing a single halogen into 2-(3,5-dibromophenyl pyrrole), but, whether or not it acts iteratively to install all 5 halogens in both the phenol and pyrrole ring in the natural system remains unclear.^151^

##### 2.2.3 Late stage halogenation by haloperoxidases

Vanadium-dependent haloperoxidases (V-HPOs) have been identified that are involved in the biogenesis of halophenol containing natural products. The napyradiomycins (including napradiomycin B1 **76**) are polyketide-terpenoid natural products identified in marine streptomycetes, over 50 members of this suite of natural products have been identified so far. V-HPOs identified within the biosynthetic gene cluster include NapH1, and NapH4. NapH1 is particularly notable, this versatile enzyme acts upon geranylated tetrahydroxynapthalene (THN) derivative, stereoselectively hydroxylating it whilst mediating regioselective chlorination (Scheme 5). NapT8 and NapH3 operate successively to catalyse prenylation and then an α-hydroxy ketone rearrangement. NapH1 then operates once more to halogenate the newly introduced prenyl unit. The reaction is most likely to proceed *via* a halonium species, which is then opened by the hydroxyl that NapH1 previously introduced, thereby enabling enantioselective cyclisation. In a similar manner, NapH4 mediates halogenation of the geranyl unit, again most likely proceeding *via* the halonium species, and promoting intramolecular cyclisation.^152^ The complex cyclic scaffolds of the merochlorins **77**, **78** are formed using similar chemical logic. Enzymatic total syntheses of napyradiomycin B1 **76** has been enabled in 18% yield.^153^

##### 2.2.4 Substrate scope: phenols

**Fig. 8:**
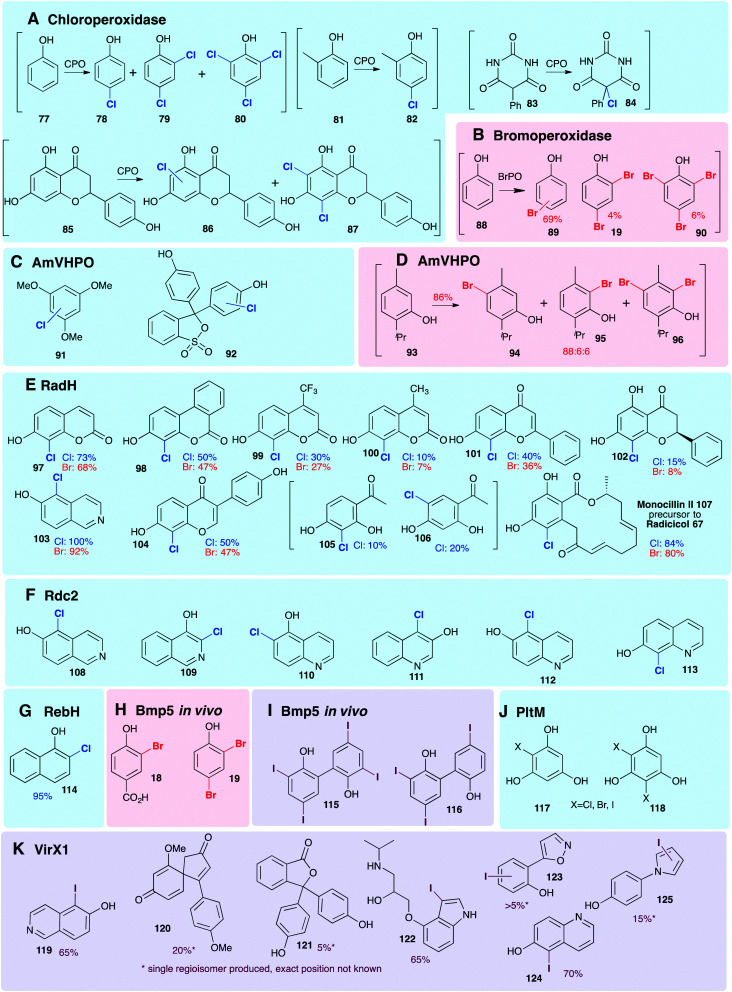
Representation of some of the diverse phenols that are accepted as non-natural substrates by different wild-type halogenases. All experiments are *in vitro*, unless specified otherwise, and % conversions reported as determined by LC/LC-MS. Enzymes include (A) chloroperoxidase (heme iron-dependent),^156–158^ (B) bromoperoxidase (vanadium-dependent)^159^ (C and D) AmVHPO (vanadium-dependent)^107^ (E) RadH (FDH) chlorination;^144^ (F) Rdc2 (FDH) chlorination and bromination;^143,160^ (G) RebH (FDH) chlorination^126^ (H and I) Bmp5 brominase (FDH) *in vivo*.^45^ (J) PltM (FDH) associated with transcriptional regulation of pyoluteorin **43** biosynthesis,^51,161^ (K) VirX1 (FDH from a virus, the first FDH with a preference for iodination).^47^ Chlorination, bromination and iodination are colour coded blue, red and purple respectively.

Smaller, freestanding, non-native phenolic substrates have been demonstrated to be processed by variant A FDHs and HPOs (Fig. 8), revealing these enzymes to be useful biocatalysts. The employment of the HPOs, in this context, whilst enabling halogenation of sterically bulky substrates, results in mixtures of regioisomers and polyhalogenated compounds. This is a direct result of most HPOs releasing free hypohalous acid; in this context, the enzyme has little control over the regiochemistry of the transformation, and it is simply the most electron-rich region(s) of the substrate that are halogenated. In contrast, the FDHs usually accept much smaller substrates but are generally highly regioselective. PltM has been shown capable of mono **117** or di-halogenation **118** of phloroglucinol (Fig. 8J), as well as being able to accept resveratrol as a substrate.^51^ Wild type RebH, GsfI, and ThaI have been shown capable of processing a broad range of non-natural substrates containing phenolic moieties.^154^ Exceptions may be seen with RadH/Rdc2 and VirX1, FDHs with much more open and accessible active sites, capable of regioselectively monohalogenating much larger phenolic substrates.^47,143,144^

#### 2.3 Pyrrolic substrates

**Scheme 6:**
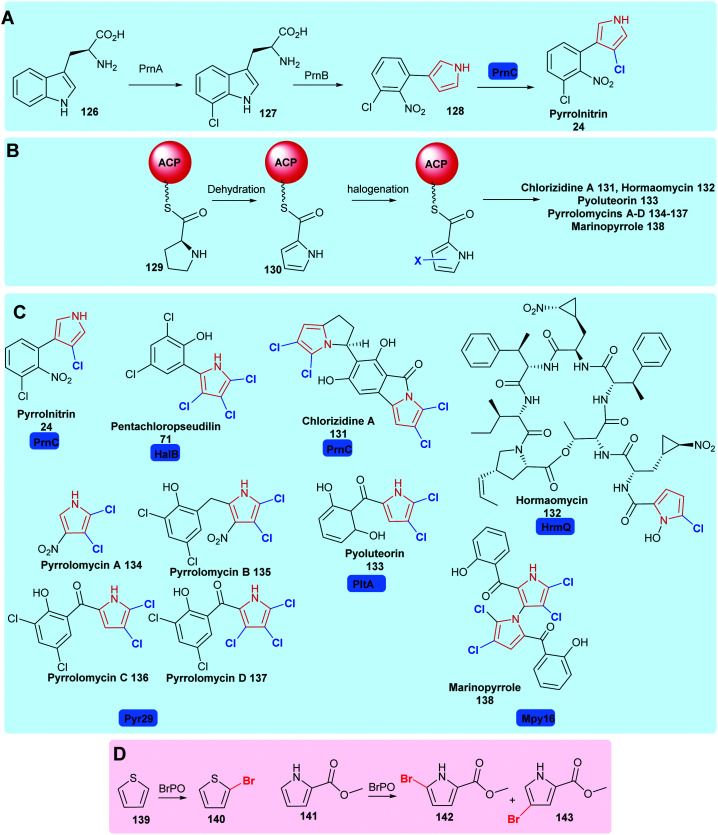
Naturally occurring halopyrrole containing compounds. (A) The biogenesis of pyrrolnitrin **24** involving a variant A FDH PrnC. (B) The enzyme tethered biosynthesis of halopyroles from proline, employing variant B FDHs. (C) Bioactive compounds containing halopyrroles including pyrrolnitrin **24**,^34^ pentachloropseudilin **71**,^151^ chlorizidine A **131**,^162^ hormaomycin **132**,^163^ pyoluteorin **133**,^164^ pyrrolomycins **134–137**^165^ and marinopyrrole **138**,^166^ noting the halogenases utilised in their construction. (D) Biocatalytically vanadium-dependent bromoperoxidases may be utilised to monobrominate thiophene and pyrroles.^167^ Chlorination is colour coded blue.

Substituted pyrroles are also widely used in medicine and agrochemistry. In nature, halogenases may be found that are capable of mono- or poly-halogenating pyrroles. Halopyrrole containing natural products include pyrrolnitrin **24**,^34^ pentachloropseudilin **71**,^151^ chlorizidine A **131**,^162^ hormaomycin **132**,^163^ pyoluteorin **133**^164^ and pyrrolomycins **134–137**^165^ (Scheme 6). PrnC, a variant A FDH, chlorinates the pyrrole of **128** in the final step of pyrrolnitrin **24** biosynthesis.^34^ Here, the pyrrole is generated through the cleavage and rearrangement of 7-chlorotryptophan **127** (Scheme 6). HalB, which shares 45% sequence identity with PrnC, is implicated in halogenating the pyrrole of pentachloropseudalin **71**.^151^ For chlorizidine a **131**, hormaomycin **132**, marinopyrrole **138**,^166^ the pyrrole is formed by oxidation of a proline uploaded onto an acyl carrier protein (ACP), the ACP tethered pyrrole is then halogenated. Bmp2 is one such variant B flavin-dependent pyrrole halogenase, mediating the intriguing tetrabromination of the PCP-bound pyrrole ring, with the introduction of the fourth bromide, to C1, apparently triggering thioesterase mediated release and decarboxylation to restore aromaticity.^48^ Curiously, the biosynthetically-related halogenase, Bmp5, employs a chemically similar strategy of decarboxylative bromination in the conversion of *p*-hydroxy-benzoic acid to 2,4-dibromophenol (see Section 1.1.2). Mpy16 from marinopyrrole **138** biosynthesis is structurally similar to Bmp2, yet stops at dihalogenation,^166^ as do Clz5^162^ and PltA^164^ in chlorazidine A **131** and pyoluteorin **133** biosynthesis, respectively. Pyr29 is also postulated to di-halogenate an ACP tethered pyrrole **130** in the biosynthesis of the pyrrolomycins,^165^ whilst HrmQ from hormaomycin **132** biosynthesis mediates monohalogenation.^163^ Structure-guided mutagenesis has been performed on wild-type Bmp2 to generate a Bmp2 triple mutant (Bmp2-TM) which proceeds no further than dibromination.^48^

#### 2.4 Aromatic substrate scope beyond indole, phenol and pyrrole

**Fig. 9:**
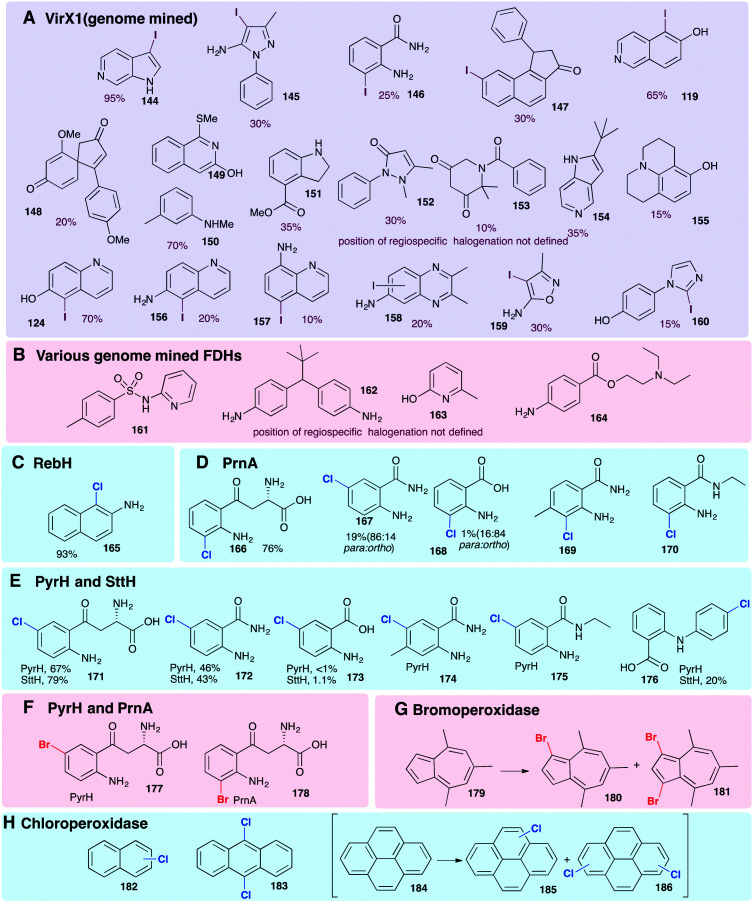
Representative selection of aromatic moieties (beyond indole, pyrrole and phenol) that may be accepted as substrates for enzymatic halogenation. Regiochemistry and conversions are given where reported in the primary literature. Enzymes include (A) genome mined VirX1, a variant A FDH with a very broad substrate scope, the first halogenase to be isolated from a virus and the first FDH to show preference for iodination.^47^ Notably, the regioselective halogenation of a diverse range of both small and large substrates may be seen including several less electron-rich and less activated substrates (bromination of all substrates also possible). (B) A series of genome mined FDHs, again, halogenation of sterically more bulky substrates may be seen (bromination of all substrates also possible).^154^ (C–F) Anilines and anthranilates processed by variant A FDHs RebH, PrnA, PyrH and SttH, respectively.^79,80,126^ (G) Vanadium-dependent bromoperoxidase capable of brominating bulky substrates.^168^ (H) Heme-iron dependent chloroperoxidases shown to be capable of processing bulky, planar modestly activated compounds such as pyrenes, mixtures of regiochemistries and levels of substitution are observed.^169^ Chlorination, bromination and iodination are colour coded blue, red and purple respectively.

Though the vast majority of natural substrates for the FDHs and haloperoxidases studied so far are indolic, phenolic or pyrrolic in nature, they have been effectively employed in installing halogens into other substrate classes. Generally, once again, the FDHs are seen to confer greater regio- and substrate specificity than the HPOs that generate and release free HOX. Initial reports of wild-type flavin-dependent halogenases had been indicative of a fairly modest and narrow substrate scope. However, RebH, PrnA, PyrH, and SttH have been shown capable of processing a series of anilines including kyuneride **171**, anthranilamide to give **172** and, to a lesser extent, anthranilic acid to give **174** (Fig. 9C–F).^79,80^ These studies also gave insight into factors governing regiospecificity. With PyrH, regiospecific *para*-chlorination **171** of kynurenine was observed whereas PrnA mediated the formation of the *ortho*-chlorinated product **166** exclusively, a product that is more chemically challenging to access. PyrH could also be used to achieve the regiospecific *para*-chlorination **172** of anthranilamide, whereas use of PrnA resulted in a 86% : 14% *para *: *ortho* mixture. Mutations could be effectively employed to modulate *ortho*:*para* selectivities and improve yields for these unnatural substrates.^79^ Chlorination of naphthalen-2-amine to give **165** by RebH *ortho* to –NH_2_ is achieved in a high yield of 93%.^126^

The halogenation of sterically bulky substrates can be enabled using haloperoxidases. As there is generally no substrate binding site, and free HOX is released, the substrates that can be halogenated using these systems are not limited by size. However, as is usually expected for an HPO that releases HOX, series of regioisomers, as well as mono, di, and tri halogenated compounds result (Fig. 9G).^169^ For example, heme iron-dependent chloroperoxidase (CPO) from *C. fumago* has been used to give chlorinated analogues of naphthalene **182**, anthracene **183** and pyrenes **185** and **186**. A vanadium-dependent bromoperoxidase from the marine algae *Ascophyllum nodosum* has also been employed mono and dibromination 4,6,8-trimethylazulene **179** (Fig. 9G), though the instability of the resultant product hindered purification.^168^

To achieve regioselective electrophilic halogenation, generally FDHs are needed, however, historically these have shown fairly narrow substrate scope. In 2019, new FDHs were revealed that had been found using *in silico* genome mining approaches (Fig. 9A and B). These halogenases were able to accept much larger substrates and showed a much broader substrate scope than any FDHs examined previously. VirX1, with a preference for iodination, was demonstrated to be capable of regiospecifically iodinating or brominating a diverse portfolio of substrates with a variety of steric and electronic demands, and some of which might be considered to be only weakly activated. Good conversions of a wide range of unnatural substrates are possible with this unusual enzyme from a virus.^47^ Similar infomatics-led approaches have been utilised by the Sewald and Lewis groups, further demonstrating that natural FDHs do exist with broader substrate scope.^170^

#### 2.5 Aliphatic substrate scope for biocatalytic halogenation

**Scheme 7:**
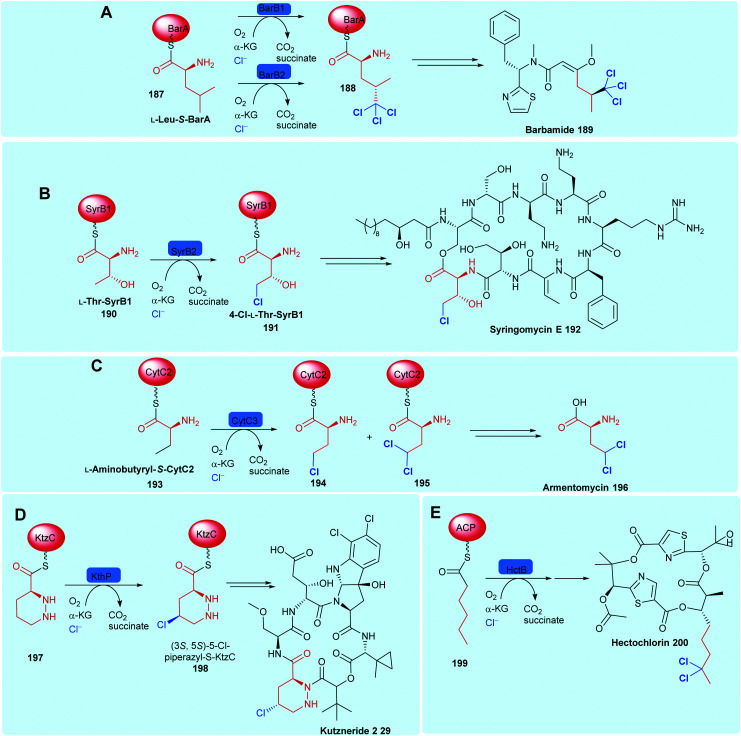
Bioactive compounds where installation of halogen on a protein-tethered aliphatic substrate is performed selectively by a NHFeHal, including (A) barbamide **189**,^60^ (B) syringomycin E **192**,^58^ (C) armentomycin **196**,^171^ (D) kutzneride 2 **29**^118^ and (E) hectochlorin **200**.^172^

The NHFeHals functionalise unactivated sp^3^ carbons. The selective C–H activation that these enzymes are capable of is challenging to achieve using even modern synthetic methodologies. Prior to 1998, these radical halogenases were unknown, however, the observation that the cyanobacterial natural product, barbamide **189**, contained a leucine residue with one trihalogenated methyl group, led Willis and co-authors to postulate that the enzymes involved in its biosynthesis perhaps proceeded *via* a radical mechanism.^173^ Over half a decade later, SyrB2, from the biosynthesis of syringomycin E **182**^46^ by *Pseudomonas syringae* B301D, was the first member of this new family of halogenases to be biochemically and structurally characterised (Scheme 7).

**Fig. 10:**
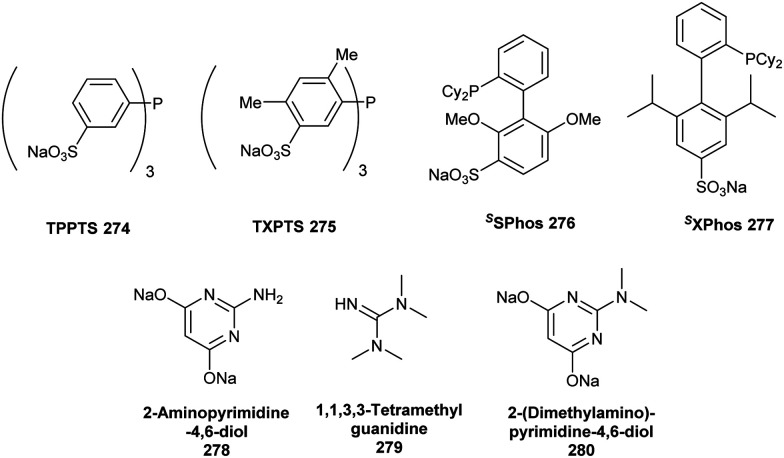
Selected water-soluble ligands used for aqueous Suzuki–Miyaura cross-coupling reactions in the presence of halogenase enzymes or even living systems.

**Scheme 8:**
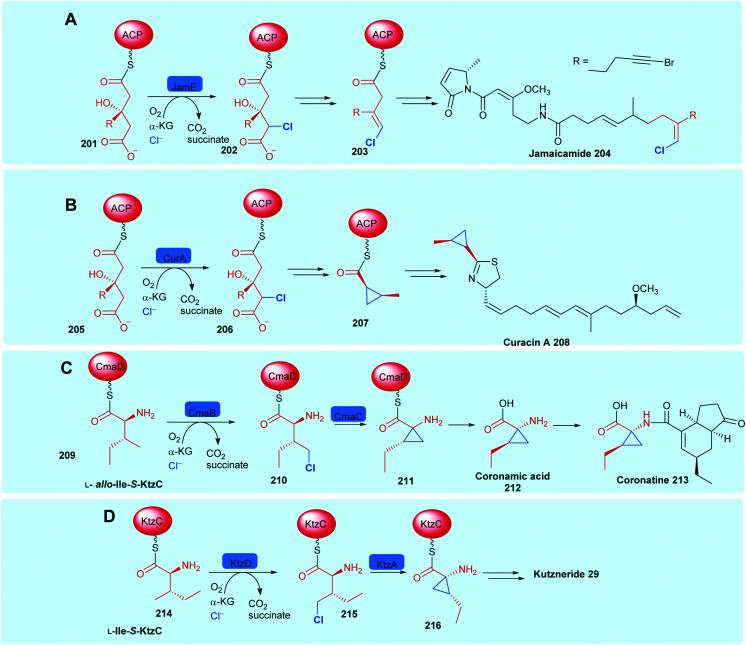
Installation of halogen on protein-tethered aliphatic substrate performed selectively by a NHFeHals, in the biosynthesis of natural products, enabling alkene generation and cyclopropane generation. (A) Jamaicamide **204**,^61^ (B) curacin A **208**,^174^ (C) coronatine **213**,^175^ and (D) kutzneride **29**.^118^ Notable similarities may be observed between the first steps in jamaicamide, vinylchloride formation and curacin cyclopropyl biosynthesis. Coronamic acid and kutzneride **29** biosynthesis are initiated by the halogenation of the γ-methyl of different diastereoisomers of l-isoleucine.

Like the FDHs, the NHFeHals may be subdivided into those that operate only on protein tethered substrates (which we call here the variant B NHFeHals, to be consistent with the variant B FDHs that also operate only on protein tethered substrates), and the perhaps more biotechnologically useful, and recently discovered variant A NHFeHals that process free substrates (see Section 1.2). The variant B type NHFeHals have proved challenging to handle requiring anaerobic conditions, nevertheless they catalyse series of intriguing chemistries, including cryptic halogenation *en route* to the installation of cyclopropyl or alkynyl motifs, notable examples may be seen in jamaicamide **204**,^61^ curacin A **208**,^174^ coronatine **213**^175^ and kutzneride **29** biosynthesis (Scheme 8).^118^ The need for covalent substrate tethering prior to halogenation means that variant B halogenases have less biotechnological potential at the current time, and will be discussed just briefly in the following section.

##### 2.5.1 Tethered substrates with unactivated sp^3^ carbon centres

NHFeHals capable of processing a variety of very different tethered substrates are known and have been shown to be capable of selectively functionalising terminal methyl groups and internal methylenes. Tri-chlorination of the pro-*R* methyl of peptidyl carrier protein (BarA) tethered leucine **187** by tandem action of BarB1 and BarB2,^60^ and di-chlorination and mono-chlorination of the γ-methyl of peptidyl carrier protein tethered l-2-aminobutyric acid **193** and l-threonine **190** by CytC3 and SyrB2 has been demonstrated through *in vitro* reconstitution experiments by Walsh and co-workers (Scheme 7).^58,171^ CytC3 and SyrB2, have a level of similarity (58% identity, 71%) similarity and process these subtly different substrates. Beyond amino acids, NHFeHals are known that can process tethered piperazines, fatty acids and tethered polyketide intermediates. Examples include, KthP, which mediates the generation of the 5-chloropiperazyl motif in kutzneride **29** biosynthesis,^118^ and HctB responsible for chlorination at C5, of an acyl-carrier protein tethered hexanoate **199**, in the biosynthesis of the antifungal hectochlorin by cyanobacterium *Lyngbya majuscula* (Scheme 7).^172^

##### 2.5.2 Unactivated sp^3^ carbon centres, processed as free, non-tethered substrates: enzymatic generation of alkyl halides

**Scheme 9:**
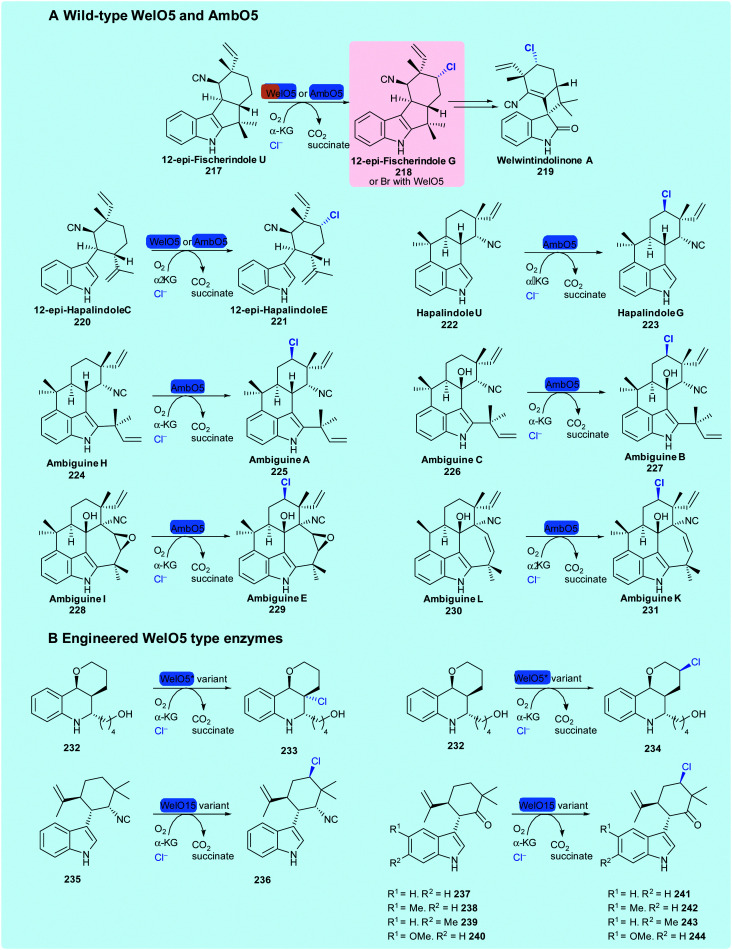
Known substrate scope of wild-type NHFeHals WelO5^62^ and AmbO5^83^ and engineered varieties of WelO15^178^ and WelO5*.^179^

The discovery of WelO5 from *Hapalosiphon welwitschii* UTEX B1830, the welwintindolinone A **219** producer, by Liu and coworkers was ground-breaking; for the first time, a NHFeHal could be seen to process non-enzyme tethered substrates. Its regioselective and stereoselective halogenation of a complex carbocycle, renders it an exciting tool for biotechnological applications. WelO5 monochlorinates 12-epi-fischerindole U **217** and 12-epi-hapalindole C **220** (Scheme 12),^62^ and has been demonstrated to enable bromination of the same substrates, leading to their enhanced antibacterial activity.^176^ Sharing 79% sequence identity with WelO5, AmbO5 was subsequently discovered through the analysis of the ambiguine **53** biosynthetic gene cluster.^83,177^ AmbO5 accepts a wider scope of substrates than WelO5 and selectively chlorinates a range of fischerindole (**52**, **53**), hapalindole (**222**) and ambiguine alkaloids (**56)** (Scheme 9A).^83^ By generating hybrid enzymes consisting of the N-terminus of WelO5 and C-terminus of AmbO5 (and vice-versa), some success of halogenating a slightly wider scope of hapalindole type molecules was achieved.^83^

Initial sequence and mutational analysis of WelO5 and AmbO5 pointed to eleven C-terminal residues, likely influencing substrate utilisation.^180^ The potential to develop WelO5 through directed evolution to improve the biocatalytic capabilities and extend substrate scope has been attempted by a number of groups. Hoebenreich and coworkers used structure-guided direct evolution to develop variants of WelO5 homologue Wi-WelO15 from *Westiella intricata* HT-29-1 capable of selectively chlorinating non-natural hapalindole **235**, and hapalindoles **237–240** containing a ketone moiety at the position of the natural isonitrile.^178^ Buller and coworkers engineered variants of another homologue WelO5* for selective halogenation at two separate positions on a martinelline analogue **232**,^179^ representing the first successful biocatalytic installation of a halogen by WelO5 type enzymes on a substrate that is notably different to their natural hapalindole targets. Both Buller and Hoebenreich identified amino acid positions, which seem to play an important role in the regioselectivity of these enzymes towards non-natural substrates, opening up possible opportunities for rational re-engineering of these catalysts.

The co-crystal structure of WelO5 and native substrate was used in a structural search for other enzymes capable of alkyl halogenation of different substrates. In this manner, the hydroxylase SadA was identified. Minor modification to the active site and coordination of the FeII (D157GSadA) resulted in an enzyme capable of chlorinating as well as hydroxylating its natural substrate.^182^

**Scheme 10:**
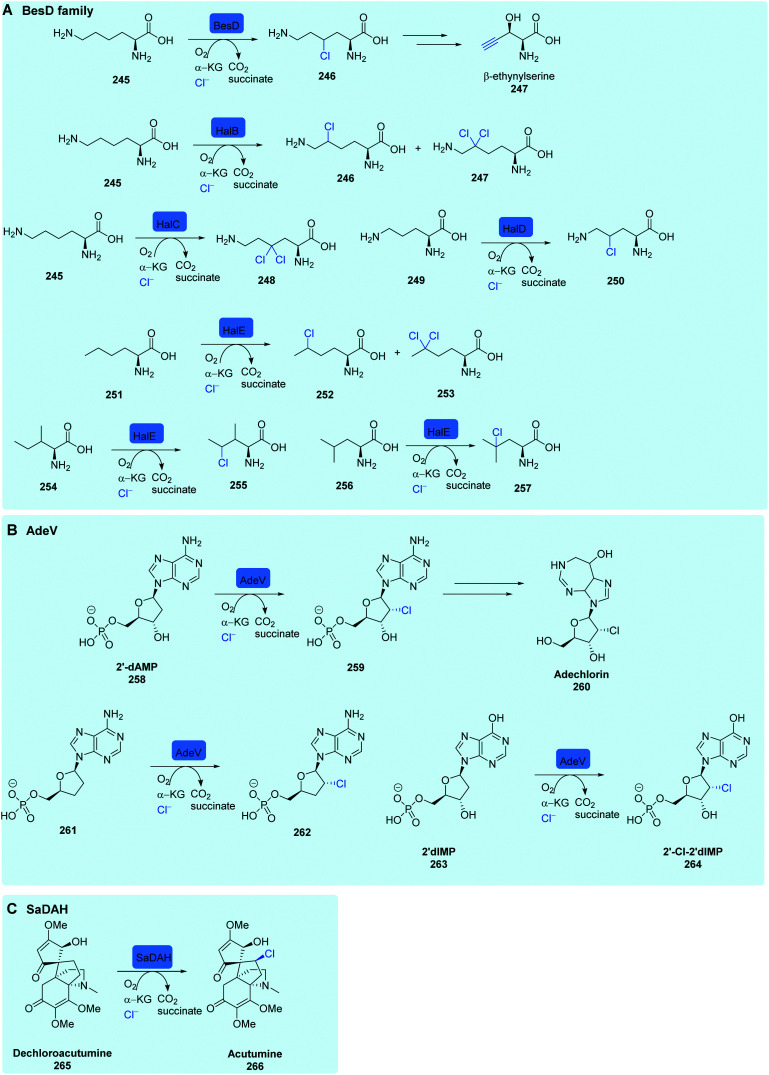
NHFe halogenases. (A) Known substrate scope of the NHFe halogense BesD and enzymes from the BesD family,^84^ (B) AdeV chlorination of 2′-dAMP in adechlorin biosynthesis and other known substrates of AdeV,^85^ (C) SaDAH chlorination in acutumine biosynthesis.^181^

A second series of variant A NHFHals that operate on substrates that are not covalently tethered to a carrier protein (free substrate NHFe halogenases) have recently been discovered, and named the BesD family. Like CurA, CmaB, KtzD (Scheme 8) and JamE, BesD is a cryptic halogenase, and was discovered in the biosynthetic pathway of the amino acid β-ethynylserine **247** (βes) *from S. cattleya*, after knockouts of non-essential amino acid desaturases pointing towards a novel enzymatic production of the alkyne moiety.^183^ Purified BesD was shown to selectively chlorinate free lysine **245**, producing 4-Cl lysine **246**.^84^ Subsequent C–C cleavage and elimination of the installed chlorine was shown to lead to the terminal alkyne (Scheme 10A). BesD has low sequence identity to both substrate bound SyrB2 (7%) and variant B prototype WelO5 (11%), instead having a much higher identity to predicted hydroxylases (>46%). Rather than being a standalone enzyme, BesD was shown to be part of a variant A NHFeHals.^84^ Homology networks were produced from hits arising from a sequence-based homology search of BesD. This approach resulted in the discovery of 20 more halogenases that act on free amino acid substrates. The range of substrates was diverse, with BesD and other enzymes accepting lysine but also ornithine, while the hydrophobic amino acids leucine, isoleucine, and norleucine, were shown to be substrates for PrHalE (Scheme 10A). A selection of these halogenases was shown to perform alongside amino acid metabolising enzymes to produce chlorinated heterocycles, diamines, and α-keto acids from lysine (SwHalB), ornithine (PkHalD), and norleucine (PrHalE). Remarkably, all enzymes retained fidelity for halogenation over hydroxylation, while the radical halogenase HalB (different to the FDH HalB) from *Streptomyces wuyuanensis* (SwHalB) was able to accept bromine and azide anions to produce bromo-lysine and azido-lysine. Chlorolysine, generated by SwHalB and PfHalA, could also be incorporated into a 9 amino acid peptide using an *in vitro* transcription and translation system-suggesting a potential use for these enzymes in production of more complex natural product analogues.^84^

Recently, variant A NHFHal capable of halogenating nucleosides, named AdeV, has been discovered in the biosynthesis of the chlorinated natural product adechlorin **260** in *Actinomadura* sp. ATCC 39365, opening up the path to access valuable halonucleosides^85^ (Scheme 10B). AdeV was shown to have 15% similarity to WelO5. Gene knockout experiments confirmed its role in adechlorin biosynthesis. AdeV acts in the early stages of biosynthesis on free nucleoside 2′-deoxyadenosine-5′-monophosphate (2′-dAMP) **258** to generate 2′-Cl-2′-dAMP. *In vitro* assays revealed that 2′-deoxyadenosine (2′-dA), identical to 2′-dAMP apart from the 5′- phosphate moiety, was not accepted as a substrate, indicating that the presence of this phosphate is essential for substrate binding and halogenation activity. Consistent with this logic, two other phosphorylated nucleosides 2′,3′-dideoxyadenosine monophosphate **261** and 2′deoxyinosine-5′-monophosphate (2′-dIMP) **263**, were accepted by AdeV and converted to their chlorinated counterparts indicating a level of enzyme promiscuity. However, the natural substrate 2′-dAMP showed the highest levels of conversion.

Several plant species are known to generate halogenated metabolites. The toxin fluoroacetate is produced in many plants including *Camellia sinensis* (from which tea is generated). Fatty acid derivatives of fluoroacetate such as ω-fluorooleic acid accumulating in the seeds of *Dichapetalum toxicarium*.^181^ An exciting new NHFe halogenase DAH that performs the stereoselective late stage chlorination of the complex alkaloid dechloroacutimine **265**, (Scheme 10C) produced by menispermaceae plants, has been found and its activity demonstrated *in vitro*. This represents the very first NHFeHal found in plants.^181^ Phylogenetic analysis indicates that DAH, which has a variant in *Menispermaceae canadense* (McDAH) and *Sinomenium acutum* (SaDAH), evolved independently from the previously discussed bacterial NHFeHAls, potentially being an example of parallel evolution in halogenated natural product metabolism. *In vitro* assays against a wide range of alkaloids indicated that DAH was highly selective towards its natural substrate, with no other small molecules being accepted for halogenation. As with some other halogenases, DAH was shown to accept azide anions as well as halides, and convert (−)-dechloroacutumine to 11-azido-dechloroacutumine.

**Scheme 11:**
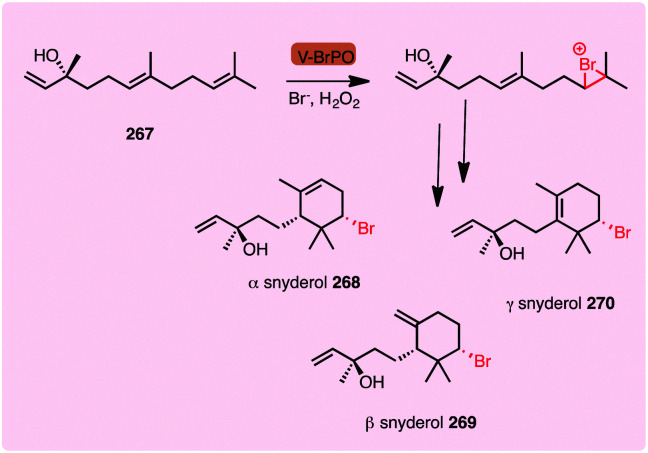
V-BrHPO catalysed terpene bromination and cyclisation event in snyderol biosynthesis.^184^

Beyond the NHFe halogenases, alkyl halides can be generated by a series of vanadium-dependent haloperoxidases. An elegant cyclisation cascade is initiated by the selective bromination of a single alkene within a terpenoid; the resultant bromonium undergoes ring opening affording the alkyl bromide. For example, the vanadium bromoperoxidase from *C. officinalis* brominates the terpenoid precursor (*E*)-(+)nerolidol **267**, yielding cyclised snyderols **268-230**.^184^ For β- and γ-snyderol, a single diastereoisomer is produced. This provides an exciting biocatalytic opportunity as synthetic methods have succeeded only in forming a mixture of two diastereoisomers of each product (Scheme 11). In a similar manner, chlorination, mediated by haloperoxidases, may be seen to generate chloronium species, initiating cyclisation cascades. Installation of chlorine onto the aliphatic moiety of SF2415B1 by NapH1 allows cyclisation of the structure to afford a cyclic ether, offering potential for synthesis of napyradiomycins **76** (Scheme 5) with cytotoxic and antibacterial properties.^155^

**Scheme 12:**
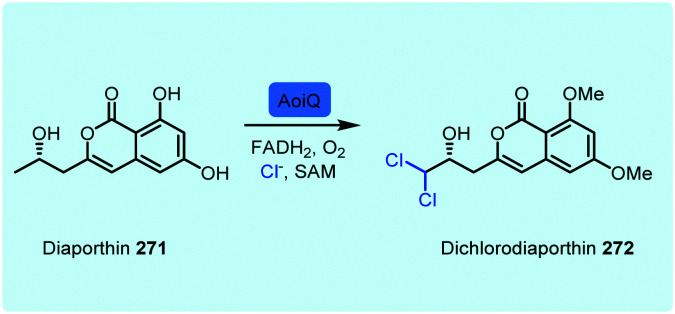
FDH AoiQ dichlorination and dimethylation of diaporthin **271**.^186^

Notably, a small number of FDHs process aliphatic compounds. One such example is CmlS, an FDH from chloramphenicol biosynthesis implicated, through *in vivo* studies, in the generation of a dichloroacetyl moiety.^185^ In this unusual FDH, the flavin cofactor is covalently linked to the enzyme. The malonyl-CoA substrate is proposed to form an enolate, stabilised through hydrogen bonding to tyrosine, this species may then react with a proximal chloramine.^44^ Beyond the halogenation of tethered malonyl-CoA by CmlS, other exciting observations of the applications of these enzymes are noted. Intriguingly, an FDH from a fungus, AoiQ, has been demonstrated to regioselectively chlorinate an unactivated terminal sp^3^ carbon of a freestanding molecule **271**, in the biosynthesis of orthosporin (Scheme 12). So far, AoiQ is unique amongst other flavin-dependent halogenases in its ability to perform this task. Although the mechanism for this reaction is unknown, it has been postulated by Hertweck and coworkers that, though it is a FDH, it may proceed *via* a radical reaction.^186^ Notably, like KrmI, it is one of the few fused halogenases to be explored. In the case of AoiQ, it contains a functional *O*-methylation domain. The mystery as to how it operates remains to be revealed.

### 3. Approaches toward improved biocatalytic halogenation, and steps toward upscaling

Considerable effort has been invested into engineering and evolving the biocatalytic activity of halogenases. Studies have predominantly focussed upon the FDHs in order to alter substrate profiles, modify regiochemistries of halogenation and enhance biocatalytic stability. Greater structural understanding of these enzymes has enabled progress to be made in their rational redesign.^187,188^ Such approaches combined with substrate walking methods^189^ have enabled the utilisation of halogenases in the selective decoration of increasingly more complex substrates, as well as stabilising enzymes to operate at higher temperatures.^190^ Engineering of the NHFeHals has also enabled their application to a broader series of substrates (see Section 2.2.4).^178,179^ Though the evolution of a small number of tryptophan halogenases for utilisation for halogenation of a broader series of substrates has proved useful, other approaches have considered the natural diversity of the halogenases. The broad range of halogenated structures that have been noted, over 5000 to date,^13^ together with the variety of organisms from which they are produced, indicates that many diverse halogenases remain to be discovered. *In silico* discovery approaches have been utilised to mine for various halogenases within uncurated genomic deposits, leading to the discovery of more substrate flexible halogenases from diverse sources including viruses.^47,170^ Discovery of novel halogenases remains at the forefront of biocatalytic halogenation research. For effective biocatalysis, good amounts of soluble protein are required. The Lewis group have effectively improved heterologous production of RebF through including a cleavable maltose binding protein (MBP) tag, to enhance solubility of the enzyme, and co-expressing both the halogenase and the concomitant reductase, RebF with the chaperonins GroEL/ES, in addition to developing a cofactor recycling system.^126^ Advances on FADH_2_ regeneration have recently been achieved by replacing NADH with synthetic mimics.^191^ A photocatalytic approach has been successfully applied to the VHPOs. The Gulder group cleverly demonstrated that by illuminating their biocatalytic system with 455 nm light, and including sacrificial electron donors, cofactor recycling could be addressed, and a ready supply of reduced cofactor afforded in a relatively inexpensive manner.^192,193^ An approach that has made a significant difference to the utilisation of FDHs is the application of cross-linked enzyme aggregates (CLEA). FDHs generally have poor stability, impacting their catalytic longevity and utility in scaled up reactions. The robust nature and solvent tolerance of Cross-Linked Enzyme Crystals was first reported in the early 1990s, but the technique required the enzymes of interest to first be crystalised.^194^ The application of CLEA was first demonstrated for PenAcylase, an aminoacylase utilised in the generation of ampicillin, where CLEA were generated from precipitating, rather than crystallising, the enzyme prior to cross-linking.^195^ By using a CombiCLEAS approach of 1 g of l-tryptophan to 1.8 g of l-7-Br-tryptophan could be achieved with complete conversion within an 8 days period.^130^ To achieve this, the lysate from *Escherichia coli* (*E. coli*) (6 L), that had been grown with a RebH expression vector, was combined with PrnF, the flavin reductase from *Pseudomonas fluorescens*, and an alcohol dehydrogenase from *Rhodococcus* sp., that had been precipitated with ammonium sulfate, was then cross-linked with 0.5%/wt glutaraldehyde to form the CombiCLEAS.

### 4. Site selective C–H activation through hyphenation of enzymatic halogenation and synthetic diversification

The opportunity to combine enzymatic halogenation with chemical modification, in particular catalytic cross-coupling, is exciting and provides a much-needed tool for molecule constructions. This concept, first reported in 2010, with the tryptophan halogenase PrnA being utilised to give access to a new to nature haloindolic natural product, which was subsequently functionalised through Suzuki–Miyaura cross-coupling,^196^ has been gaining momentum. This section comprehensively reviews the current state-of-the-art and reflects on technologies enabling the hyphenation.

#### 4.1 Aqueous compatible cross-coupling methodologies

Palladium-catalysed cross-coupling reactions play a central role in organic chemistry. Their utility in the formation of C–C and carbon–heteroatom bonds under mild conditions with a wide range of organic halide and nucleophilic reagents, has led to them being one of the most utilised series of reactions in the pharmaceutical industry.^197^ As with almost all enzymes, the halogenases require an aqueous environment for effective operation. To effectively couple enzymatic halogenation with synthetic diversification, aqueous compatible reaction conditions are generally required. Palladium-catalysed cross-coupling reactions have largely utilized traditional organic solvents. Standard conditions for these coupling methodologies typically use a mixed organic/aqueous base solvent system, hence the development of water solubilising ligands have been highly enabling. The first example of a water-soluble palladium catalyst for cross-coupling reactions being reported by Casalnuovo,^198^ and significant effort has been devoted to the development of hydrophilic palladium/ligand complexes for aqueous-phase catalysis since this seminal work, as well as active hydrophobic catalysts and ligand-free species based on palladium nanoparticles.^199–203^ The development of mild cross coupling conditions for the modulation of challenging and or functionally sensitive compounds is advancing, enabling the diversification and tuning of small molecules, natural products and biomolecules including nucleic acids and peptides. To this end, aqueous compatible and water soluble ligands such as TPPTS **274**,^199^ and the sterically demanding and electron-rich TXPTS **275**,^199^^s^SPhos **276**,^204^^*S*^XPhos **277**^204^ and disodium 2-aminopyrimidine-4,6-diol **278**^205–207^ have proved valuable (Fig. 10). Additionally, nitrogen based ligands enabling aqueous compatible cross-couplings such as tetramethylguanidine **279** and 2-(dimethylamino)-pyrimidine-4,6-diol **280**, both reported to have low cell toxicity, are beginning to facilitate tandem halogenation and cross-coupling strategies in living systems.^208^

**Scheme 13:**
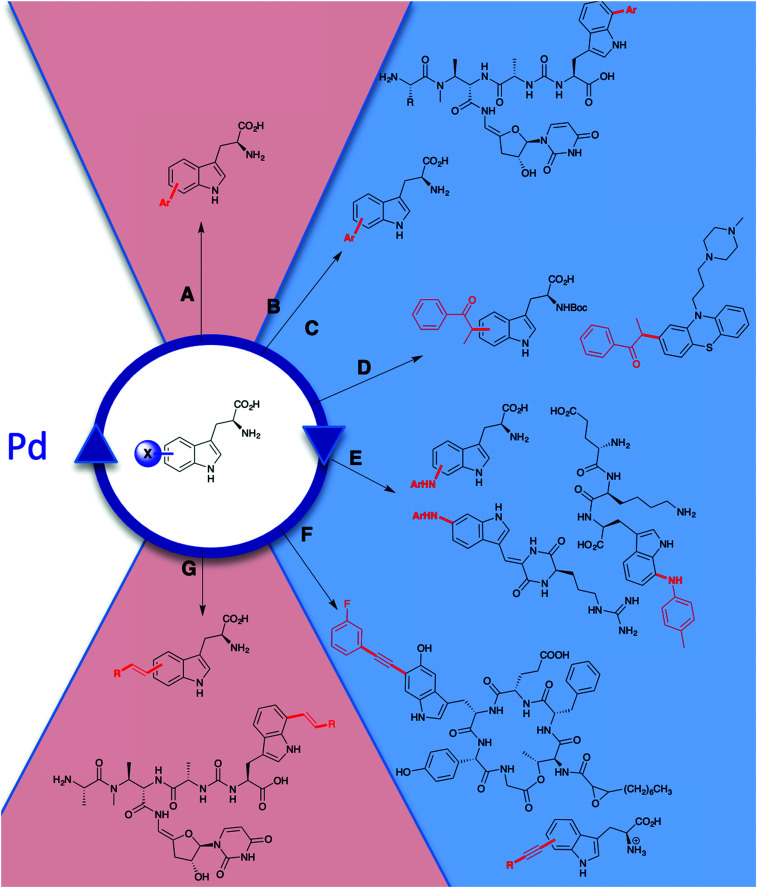
Mild aqueous conditions enabling the cross-coupling of free unprotected halotryptophan and halotryptophans embedded into natural products, biomolecules and bioactive compounds. Colour code: Blue background: conditions enable processing of aryl-chlorides in addition to more reactive aryl-bromides, Red background: least activated aryl halide processed is Ar–Br under these conditions (broad substrate scope and extension of chemistry to other systems not shown here). (A) Suzuki–Miyaura cross-coupling on bromotryptophans: phenylboronic acid (1.1 equiv.), Na_2_PdCl_4_ (2.5 mol%), TXPTS (25 mol%), K_2_CO_3_ (5 equiv.), water, 40–80 °C, 70–90% isolated yields.^209^ (B) Suzuki–Miyaura cross-coupling on chlorotryptohans, and 7-chlorotryptophan pacidamycin: phenylboronic acid (1.5 equiv.), Na_2_PdCl_4_ (5 mol%), SPhos (10 mol%), K_2_CO_3_ (5 equiv.), water:acetonitrile 80 °C, on purified material and crude extract 60–70% isolated yields.^217^ (C) Suzuki–Miyaura cross-coupling of bromotryptohans, *p*-tolylboronic acid (3 equiv.), Pd(OAc)_2_-[2-amino-pyrimidine-4,6-diol]_2_ (5 mol%), K_2_CO_3_ (6 equiv.), 45 °C, 54–85% isolated yields and 7-bromotryptophan pacidamycin in living cultures: reaction carried out in optimised culture medium at 37 °C.^206^ (D) Ketoarylation of *N*-Boc-bromotryptohans, and small water-soluble chloro and bromo pharmacophores: ketone (4 equiv.), [Pd(DtBPF)Cl_2_] (DtBPF = 1,1′-bis(di-*tert*-butylphosphino)ferrocene) (2 mol%), NaOH (4 equiv.), dioxane : water 1 : 1, 60 °C, 45–94% isolated yields.^212^ (E) Buchwald–Hartwig diversification of chloro and bromotryptophans, peptides containing bromotryptophan and the bromotryptophan containing natural product barettin: aniline (2 equiv.), [Pd(^*t*^Bu-XPhos)G1] (5 mol%), KOH (4 equiv.), dioxane : water 1 : 1, 100 °C, 8–48 min, 31–75% isolated yields.^213^ (F) Sonogashira diversification of bromotryptophans, peptides containing bromotryptophan and the new to nature bromotryptophan containing natural product cystargamide: alkyne (1 equiv.), [PdCl_2_(CH_3_CN)_2_] (5 mol%), sXPhos (15 mol%), Cs_2_CO_3_ (2.5 equiv.), acetonitrile : water 1 : 1, 100 °C, 25–97% isolated yields.^211^ (G) Heck diversification of bromotryptophans, peptides containing bromotryptophan 7-bromotryptophan containing pacidamycin: alkene (1.5 equiv.), Na_2_PdCl_4_ (10 mol%), TXPTS (23 mol%), Na_2_CO_3_ (4 equiv.), acetonitrile : water 1 : 3, 90 °C, 51–95% isolated yields.^210^

Specifically, with the goal of enabling C–H activation through enzymatic halogenation and chemical cross-coupling, our team has developed series of aqueous cross-coupling procedures, enabling the cross-coupling to be compatible with molecules decorated with sensitive functional groups, as well as with enzymes and even in live cell environments. We have initiated our development of these catalytic conditions by focussing on challenging free and unprotected halotryptophans. Our choice has been directed by three factors:

– The availability of a well-characterised series of tryptophan halogenases that may be utilised in standalone format in conjunction with a biosynthetic pathway to access halotryptophans and synchronously new-to-nature halotryptophan containing natural products;

– The utility of tryptophan as a fluorescence tag and a moiety that governs protein folding, and the attraction to being able to tune these properties;

– And the challenge that free tryptophan confers, rendering it a suitably stimulating test bed. We reasoned that by developing conditions that were suitable to address the cross-coupling of free halotryptophan, a small molecule with the propensity to coordinate to the catalyst and render it inactive, such conditions could be readily applied to other more tractable moieties. To this end, we developed a range of aqueous conditions enabling the Suzuki–Miyaura,^209^ Heck,^210^ Sonogashira,^211^ keto-arylation^212^ and Buchwald–Hartwig^213^ diversification of free unprotected tryptophan, peptides, and natural products (Scheme 13).

The development of mild aqueous chemistries has facilitated chemo-enzymatic and GenoChemetic approaches. Site selective C–H activation is enabled by a halogenase and synthetic diversification carried out, in some cases synchronously, through aqueous compatible Pd mediated catalysis. More reactive aryl iodides and bromides can be coupled under much milder conditions than their less activated chloro counterparts.

#### 4.2 Chemo-enzymatic approaches

**Scheme 14:**
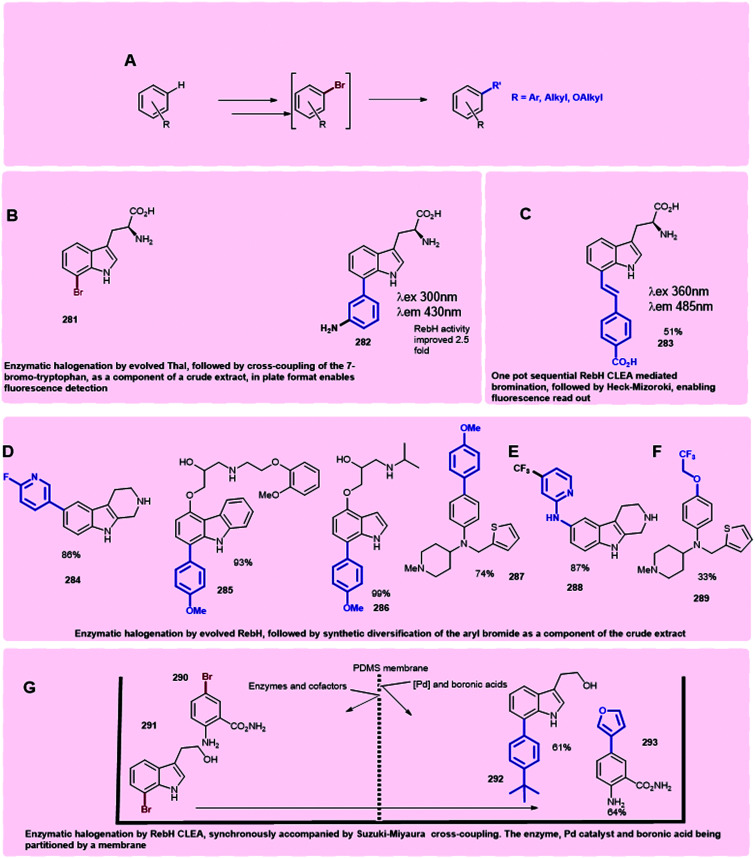
Hyphenation of halogenation and cross-coupling to achieve C–H activation, (A) general concept in which the halogenase operates first, and then the cross-coupling is carried out on the halogenated material as a component of the crude extract. This is carried out stepwise in (B–F), enabling access to a broad series of compounds, and synchronously in G where the halogenation and cross-coupling events are spatially separated through application of a membrane. (B) Bromination of l-tryptophan by Thal, followed by Suzuki–Miyaura cross-coupling of the brominated product as a component of the crude lysate.^215^ The product of cross-coupling (*λ*_ex_ 300 nm/*λ*_em_ 430 nm) is used in screening the directed evolution of RebH, resulting in a 2.5-fold increase in enzymatic activity. Cross-coupling conditions: 3-aminophenylboronic acid (10 equiv.), Na_2_PdCl_4_ (50 mol%), ^*S*^Sphos (150 mol%), K_3_PO_4_ (15 equiv.), 95 °C.^215^ (C) One-pot bromination of l-tryptophan by RebH-CLEA, followed by Mizoroki–Heck cross-coupling, in a stepwise fashion to afford C-7 substituted 7-(4-carboxystyryl)tryptophan with a *λ*_em_ of 485 nm (*λ*_ex_ 360 nm). Cross-coupling conditions: 4-carboxystyrene (5 equiv.), PdOAc_2_ (0.1–0.2 equiv.), TPPTS (0.3–0.6 equiv.), K_2_CO_3_ (5 equiv.), water, degassed under argon, 100 °C.^189^ (D–F) Sequential enzymatic bromination by evolved RebH variants, followed by (D) Suzuki–Miyaura, (E) Buchwald–Hartwig, (F) alkoxylation of tryptophan as a component of a crude extract. Conditions D: ArB(OH)_2_ (1.5 equiv.), PdOAc_2_ (0.05 equiv.), ^*S*^SPhos (0.05 equiv.), iPrOH: phosphate buffer (170 mM, pH 8.5) 1 : 1, 90 °C. (E) ArNH_2_ (3 equiv.), PdOAc_2_ (0.03 equiv.), BrettPhos (0.03 equiv.), NaO*t*-Bu (6 equiv.), dioxane, 100 °C. (F) CF_3_CH_2_OH (2 equiv.), [(allyl)PdCl]_2_(0.005 equiv.), RockPhos (0.015 equiv.), Cs_2_CO_3_ (2 equiv.), toluene, 90 °C.^189^ (G) One-pot, synchronous halogenation and Suzuki–Miyaura cross-coupling by PDMS membrane separated RebH-CLEA and Pd catalyst. Cross-coupling conditions: PdOAc_2_ (10 mol%), 2-(dimethylamino)-pyrimidine-4,6-diol (20 mol%), aryl boronic acid (5 equiv.) and CsF (10 equiv.), r.t. overnight then 80 °C.^211^

Hyphenation of enzymatic halogenation and synthetic derivatisation has been enabled by steering, where possible, to enzymatic bromination (or even iodination) over enzymatic chlorination, employing combinations of stabilised or partitioned enzymes, and use of mild and aqueous cross-coupling methodologies. There is burgeoning interest in employing such strategies as powerful methods for late-stage diversification on ever more complex molecules. The approach is also utilised to enable a fast fluorescence assay for enzyme screening and for directed evolution. A few enzymes, such as RadH have been shown capable of halogenating 7-hydroxycoumarin. An enhancement in fluorescence occurs when 7-hydroxycoumarin (*λ*_max_ 325 nm) is converted to 8-chloro-7-hydroxycoumarin (*λ*_ex_ 386 nm/*λ*_em_ 456 nm).^144^ In most halogenation reactions, a clear change in fluorescence is not always observable, and cross-coupling chemistry has been employed to provide a fluorescence readout that may be used to assay halogenase activity. Sewald reported the utilisation of tryptophan 5-, 6- and 7-halogenases PyrH, ThaI, and RebH for bromination of l-tryptophan substrate.^214,215^ The team then employed aqueous cross-coupling conditions. To achieve cross-coupling of the bromo-tryptophan product within the crude lysate, the Pd-catalyst loading was further increased to 50 mol%. By reaction with the boronic acid of aniline, the products (3′-aminophenyl)-tryptophans were reported to have a *λ*_ex_ 300 nm/*λ*_em_ 430 nm (Scheme 14B). This technology was utilised for microtitre plate screening enabling rapid analysis of ThaI mutants that had been generated by error-prone PCR.^215^ The Sewald team also cleverly employed CLEA technology (see Section 3) to enable bromination of tryptophan by these same halogenases PyrH, ThaI, and RebH. Not only did the utilisation of enzyme aggregates afford stabilisation, but it also provided an opportunity for readily filtering off the enzymes ahead of Suzuki–Miyaura diversification of the resultant halotryptophans. By utilising a three-step approach, organic solvents required for solubilisation of several of the substrates could be used, with Boc protection being carried out in a final step, thereby easing purification.^214^ In a similar manner, the Sewald group developed a three-step one pot reaction utilising RebH and Mizoroki–Heck chemistry for the further diversification of tryptophans. Again, by using their combi-CLEA system in a stepwise manner (Scheme 14D), the harsher reaction conditions required for Heck diversification could be utilised and enabled the generation of C-7 substituted styryl-tryptophan with a *λ*_em_ of 485 nm (*λ*_ex_ 360 nm), and Stokes shift of 7159 cm^−1^.^216^

This larger Stokes’ shift renders this technology potentially very useful for molecule tagging and enzyme assay, as well as valuable for molecule diversification. Even when stabilised as CLEA, enzymes and Pd catalysts can have poor compatibility. To achieve a synchronous one-pot enzymatic halogenation and chemical cross-coupling, and to avoid the additional filtration step, Micklefield and co-workers elegantly partitioned CLEA stabilised PyrH, RebH, RadH and SttH from the Pd catalyst through use of a polydimethylsiloxane (PDMS) membrane. Whilst the brominated enzymatic product could pass through the PMDS, it was reported to be impervious to the enzymes and cofactors. The cross-coupled product, once formed, remained in the chemo-catalytic chamber (Scheme 14G).^218^

Though the majority of chemo-enzymatic approaches published so far have focussed on tryptophans and other indolic systems, this is predominantly a reflection of the most extensively studied FDHs. Through substrate walking methodology, Jared Lewis and co-workers have impressively evolved RebH to work on substrates that are chemically distinct from its natural tryptophan substrate. Utilisation of evolved halogenases enabled bromination of a diverse series of substrates including tryptolines, sterically bulky substituted anilines as well as carvediol and pindolol, two clinically utilised beta-blockers. Conditions to enable Suzuki–Miyaura, Buchwald–Hartwig amination and alkoxylation were developed and applied mostly to the chlorinated and brominated intermediates as a component of a crude extract, resulting in high yields of the respective products **284–289** (Scheme 13D–F).^189^ With the discovery of more substrate diverse halogenases, engineering of known halogenases, enzyme stabilisation and partitioning strategies and the development of milder reaction conditions, the portfolio of functionalisable systems is set to expand significantly.

FDHs are not the only halogenating enzymes to be merged with palladium catalysed cross-coupling. V-HPOs and HPOs have been successfully used in various chemoenzymatic halocyclization reactions,^219,220^ and recently this has been merged with homogenous metal catalysis for further diversification. Deska and coworkers enabled the one-pot combination of allenol halocylization using CPO from *Caldariomyces fumago* and glucose oxidase from *Aspergillus niger* (GOx) with SPhos catalysed Suzuki–Miyaura and Sonogashira coupling to diversify a range of dihydrofurans. Excitingly, a nanobiohybrid was also generated using GOx as a support for palladium nanoparticles, which led to good yields of stepwise halocyclization and Suzuki coupling.^221^

#### 4.3 GenoChemetic approaches

Whilst in an enzymatic or whole cell biotransformation where the enzymes or cells are exogenously supplemented with substrates, in a GenoChemetic approach, the substrate and tagged intermediates are fully biosynthesised by an engineered organism. GenoChemetic processes provide an expeditious route to generating series of analogues of natural products. Semi-synthesis, though a useful tool, is limited by the innate functional groups naturally present within the molecule. GenoChemetics addresses this. It involves the introduction of a gene to complement a biosynthetic pathway, such that a chemically reactive and orthogonal handle is introduced into the natural product. This handle enables synthetic diversification of the natural product. There are so far three reports of the utilisation of halogenases within GenoChemetic systems. In each of these systems, tryptophan halogenases have been employed, with RebH, PyrH, and PrnA being installed into either pathway engineered or wild-type strains enabling the production of chlorinated or brominated analogues of the complex natural product scaffolds.

**Scheme 15:**
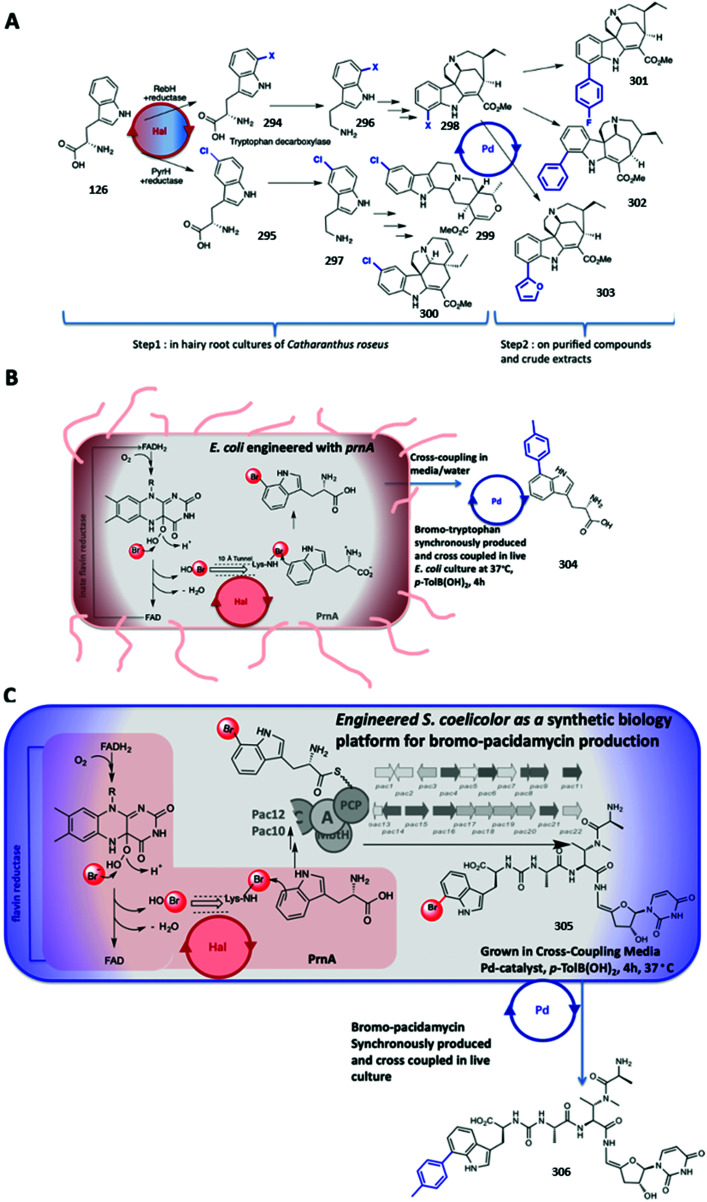
GenoChemetic approaches to C–H functionalisation of small metabolites and complex natural products. Here, a halogenase is installed to complement an existing metabolic/biosynthetic pathway, the halogenated natural product is diversified through application of cross-coupling chemistries. (A) Two step approach: hairy root cultures of *Catharanthus roseus* are engineered to include RebH, PyrH and their concomitant reductases. A series of chlorinated and brominated analogues are produced, and cross coupling has been carried out on purified compounds and on the metabolites as components of crude extracts, enabling access to a wide portfolio of functionalised molecules.^224^ Cross-coupling conditions: arylboronic acid (3 equiv.), Pd(OAc)_2_ (5 mol%), Sphos (13 mol%), K_3_PO_4_, *n*-butanol, 100 °C, 10–60 min. (B and C) One pot approach, pathways constructed in heterologous hosts, with halogenation (mediated by PrnA)^225^ and cross-coupling occurring synchronously in the presence of the living *E. coli* and *Streptomyces coelicolor*,^222^ respectively.

The halogenated metabolites have been subjected to cross-coupling chemistries. In the first example, the tryptophan halogenase PrnA was installed, through use of an integrative vector, into the genome of *Streptomyces coeruleorubidis.* It was demonstrated that there was no need to introduce PrnA's concomitant flavin reductase, as the organism's own reductases sufficed. A chlorinated analogue of pacidamycin, a uridyl peptide antibiotic, was produces at a titre of ∼1 mg L^−1^. In spite of bearing series of sensitive functional groups, this metabolite could be readily cross-coupled using the moderately mild Suzuki-Miyaura conditions that were developed. The cross-coupling was carried out both on the purified material and on material as a component of a crude extract (Scheme 15). The latter was preferable, as the more lipophilic product that resulted was much more readily amenable to purification.^217^ The generation of brominated analogues would, of course, facilitate cross-coupling under even milder conditions. However, *Streptomyces coeruleorubidis* was found to be sensitive to bromide salts, and the brominated analogue cannot readily be accessed through use of this strain. *Streptomyces coelicolor* showed good tolerance to bromide salts in a screen of series of potential heterologous expression hosts, and so the pacidamycin biosynthetic gene cluster, identified previously, was ported into this strain conferring it with the ability to produce pacidamycins. The gene encoding the halogenase PrnA was engineered into the strain and ability to produce bromo-pacidamycin was confirmed. In order to enable bromometabolite production and cross-coupling synchronously, mild cross-coupling reactions that enabled derivatisation of 7-Br-pacidamycin D **305** in the presence of the living culture were carried out. In a similar manner, *E. coli* was engineered with the ability to make bromo-tryptophan and its cross-coupling to **306** affected in the living fermentative culture.^222^ A similar system was designed in hairy root cultures of *Catharanthus roseus* in which they were engineered to include RebH, PyrH and their concomitant reductases. This led to the production of a range of chlorinated and brominated analogues of the plant's monoterpene indole alkaloid natural products **299–301**, which were successfully extracted and coupled in non-aqueous Suzuki–Miyaura couplings, either as crude extracts or purified compounds (Scheme 15A).^223,224^

## Conclusions

Though enzyme mediated halogenation was initially considered to be nothing more than an artefact or a rare event, today over 30 enzymes capable of mediating such chemistry have been structurally and biochemically characterised (Table 2), and an even greater number subject to *in vitro* and *in vivo* investigation. Halogenated compounds play a major role in the construction of molecules *via* cross-coupling methodologies, as well as making up a large percentage of medicinally and agrochemically relevant molecules. In a world that seeks more selective, greener and more sustainable solutions to molecule manufacture, halogenases hold an opportunity. They also offer potential for the vectorisation of fragments for SAR. Whilst the kinetic analyses of the halogenases is rather potted, drawing this data together is useful in identifying potential halogenases for development and use in biotransformations. Whilst the HPOs are capable of mediating halogenation of large and sterically demanding substrates, as most neither bind the substrate nor the electrophilic species, such systems generally lack regiocontrol. A few intriguing departures from this trend have been identified from marine systems, indicating that there is much more to learn about these enzymes, their operation and evolution. New variant A FDHs with reasonable catalytic efficiency and broad substrate scopes have been revealed in the last two years, enabling regioselective chlorination, bromination and even iodination on sterically and electronically demanding aromatic systems, even prior to rational redesign or evolution. Excitingly, in recent years the range of variant A NHFeHals that enable the controlled halogenation, including stereoselective halogenation, have been identified; clearly, there are many more such systems awaiting discovery. Inspired by such systems, it may be possible to generate artificial architectures that, perhaps *via* haloamines, position and deliver electrophilic halogenating agents to substrates held in the required position to affect desired regiochemistries.

Halogenation introduces a chemically reactive, chemically orthogonal moiety into a compound. This single atom modification, that can be genetically/enzymatically installed has the potential to act as a superior tag to azide or alkyne modifications. In parallel with halogenase discovery, a variety of aqueous cross-coupling reactions compatible with sensitive functionalities in natural products and biomolecules have been developed. Some of these reactions are even biorthogonal. The opportunities for building pathways and circuits of SynBio-SynChem present themselves.

## Conflicts of interest

There are no conflicts to declare.

## Supplementary Material
